# Emerging molecular mechanisms of the ECM–exosome growth-plate axis in idiopathic short stature

**DOI:** 10.3389/fcell.2026.1898879

**Published:** 2026-08-18

**Authors:** Lizhen Piao, Hong Wang, Qiuying Zhang, Jinhe Li, Hongmei Sun, Ronghe Sun, Mingming Li, Ye Wang

**Affiliations:** 1 Department of Pediatrics, China-Japan Union Hospital of Jilin University, Changchun, China; 2 Department of Pediatrics, Kailu County Hospital, Tongliao, China

**Keywords:** chondrocyte differentiation, exosomes, extracellular matrix, growth plate, idiopathic short stature

## Abstract

Idiopathic short stature (ISS) remains a clinically heterogeneous diagnosis in which impaired linear growth is often defined by exclusion rather than by mechanism. Increasing evidence suggests that ISS and related short-stature phenotypes may converge on overlapping growth-plate abnormalities characterised by disrupted chondrocyte proliferation, hypertrophic differentiation, extracellular matrix (ECM) maturation, mineralisation, and endochondral ossification. In this narrative review, we synthesize current evidence relevant to a proposed ECM–exosome growth-plate axis as a hypothesis-generating framework for ISS. The reviewed studies suggest that exosomal RNA dysregulation, ECM structural abnormalities, growth-hormone and insulin-like growth factor signalling variation, environmental and inflammatory exposures, and altered local signalling pathways may each contribute to impaired growth-plate output in specific experimental or clinical contexts. However, these diverse upstream routes appear to be associated with overlapping downstream abnormalities in chondrocyte state transition and cartilage-to-bone conversion. We propose that the cartilage ECM may function as a regulatory niche that could influence exosome diffusion, retention, uptake, spatial exposure, and RNA signalling activity within growth-plate chondrocytes. Conversely, exosomal miRNAs, lncRNAs, and circRNAs may regulate ECM synthesis, matrix remodelling, hypertrophy, and ossification. Accordingly, the axis is presented here as a hypothesis-generating model rather than as an established causal mechanism in ISS. Nevertheless, direct causal evidence remains limited, particularly regarding whether ECM composition, stiffness, proteoglycan density, collagen organisation, or mineralisation state governs exosome-mediated RNA delivery in human growth-plate models. Future studies should combine paediatric cohorts, human chondrocytes, growth-plate organoids, hPSC-derived cartilage systems, engineered exosomes, and ECM perturbation-rescue experiments to validate this axis. Defining ECM–exosome signatures may enable mechanism-based ISS subclassification, prediction of GH responsiveness, liquid-biopsy biomarker development, and growth-plate-targeted therapeutic strategies. Overall, the proposed ECM–exosome growth-plate axis may help frame ISS as a potentially molecularly stratifiable disorder involving chondrocyte state-transition failure, rather than solely as an endocrine diagnosis of exclusion.

## 1 Introduction

Idiopathic short stature (ISS) remains one of the most common and biologically ambiguous causes of impaired linear growth in children. Traditionally, ISS is defined by auxological criteria in the absence of systemic disease, endocrine deficiency, malnutrition, or recognizable skeletal dysplasia. However, this exclusion-based definition is increasingly insufficient, as advances in genomics, growth-plate biology, and biomarker discovery suggest that many children classified as having ISS may harbour subtle disturbances in growth-plate regulation, extracellular matrix (ECM) biology, non-coding RNA signalling, or growth hormone responsiveness (Paparella et al., 2025; Saltarelli et al., 2022).

Postnatal linear growth depends on the coordinated activity of growth-plate chondrocytes, which drive longitudinal bone elongation through endochondral ossification. This process requires orderly transition of resting-zone progenitors into proliferative chondrocytes, followed by hypertrophic differentiation, ECM remodelling, vascular invasion, and replacement of cartilage by bone (Ağirdil, 2020; Cho et al., 2024). Recent single-cell and functional studies have refined this model by identifying distinct growth-plate stem-like populations, chondrocyte maturation programmes, and gene networks that regulate skeletal growth and height-associated traits (Baronas et al., 2023; Chu et al., 2026; Trompet et al., 2024). These findings support a shift from viewing ISS solely as a nonspecific endocrine phenotype toward considering it as a disorder of growth-plate cellular state regulation.

The ECM plays a central role in coordinating chondrocyte differentiation and growth-plate maturation. Beyond providing structural support, the cartilage ECM supplies biochemical, mechanical and spatial cues that regulate chondrocyte proliferation, columnar organisation, hypertrophy, and mineralisation. Genetic or acquired disruption of ECM components, including aggrecan, collagens, proteoglycans, matrilins, and cartilage oligomeric matrix proteins, can impair growth-plate architecture and cause short stature or skeletal dysplasia (Bendre et al., 2025; Saltarelli et al., 2022; Taieb et al., 2023). ECM abnormalities may therefore represent one biological route through which children previously classified as having ISS develop reduced growth velocity, premature growth failure, or altered responsiveness to growth-promoting therapies.

In parallel, exosomes and related extracellular vesicles (EV) have emerged as important mediators of intercellular communication in skeletal tissues. These vesicles can transfer non-coding RNAs, proteins, lipids and other regulatory cargoes to recipient cells, thereby influencing gene expression and cellular phenotype. In the growth plate, extracellular vesicle-mediated RNA transfer is particularly relevant because chondrocyte behaviour depends on tightly coordinated local communication within an avascular, ECM-rich cartilage environment (Dai et al., 2020; Lin et al., 2016; Thakore and Delany, 2025). At present, these studies support exosome-related effects on chondrocyte behaviour, but they do not directly demonstrate that growth-plate ECM properties regulate exosome uptake or cargo activity in ISS.

Despite these advances, ECM-focused and exosome-focused studies in ISS have largely developed in parallel. ECM research has examined how matrix composition, structure, and mechanics regulate chondrocyte behaviour, whereas exosome research has focused on how circulating vesicular cargoes may alter growth-plate signalling. The possible bidirectional interface between these systems remains poorly defined, including how growth-plate ECM composition, proteoglycan density, collagen organisation, matrix stiffness, and zonal architecture regulate exosome trafficking, uptake, and cargo function, and how exosomal signals may in turn remodel ECM gene expression, matrix maturation, and chondrocyte–matrix interactions. Throughout the review, findings directly supported by experimental or clinical studies are distinguished from proposed mechanistic links that require future validation.

## 2 Methods and literature approach

This structured narrative review synthesises evidence on the possible interplay between the ECM and exosome-mediated RNA signalling in ISS. The narrative review design was selected because the topic integrates mechanistically diverse evidence from growth-plate biology, ECM-defect entries, exosomal RNA studies, endocrine signalling, environmental/inflammatory exposure and translational biomarker research (Grant and Booth, 2009; Green et al., 2006). We therefore adopted a structured narrative-review framework rather than a systematic or scoping-review framework. To support this structured narrative synthesis, we searched peer-reviewed publications from 2010 through March 2026 using PubMed, Web of Science and Google Scholar. The search strategy was used to improve transparency and breadth of coverage, but it was not prospectively registered, was not conducted according to PRISMA or PRISMA-ScR, and did not involve duplicate independent screening or formal risk-of-bias appraisal. These features distinguish the present review from a systematic review or scoping review. Reporting was guided by principles for transparent narrative reviews, including clear justification of the review’s importance, description of search strategy, balanced presentation of evidence and critical interpretation of mechanistic findings.

Search terms combined concepts of “idiopathic short stature,” “growth plate,” “extracellular matrix,” “exosomes,” “extracellular vesicles,” “microRNA,” “long non-coding RNA,” “circRNA,” “growth hormone/insulin-like growth factor” and “environmental/inflammatory exposure.” We considered English-language peer-reviewed publications from January 2010 to March 2026 that could inform the conceptual question, including original clinical, translational and mechanistic studies, together with selected reviews used for contextual framing. Reference lists of retrieved articles were screened for additional sources. Records were not prioritised as core evidence when they were unrelated to ISS or linear growth, did not address growth-plate biology, ECM regulation, extracellular vesicles/exosomes, RNA signalling, growth-hormone and insulin-like growth factor (GH–IGF)-axis biology or environmental exposures, or consisted of conference abstracts, editorials, commentaries, case reports without mechanistic relevance, non-peer-reviewed sources or duplicate records. The search and selection process was summarised using article counts. Database and reference-list searches identified 535 records; after relevance-based title/abstract review, full-text checking where needed and removal of records outside the conceptual scope (489), 46 articles were selected for structured narrative synthesis (Supplementary Table S1). Selected studies were grouped according to their primary evidence role: clinical ISS studies, growth-plate or ECM mechanistic studies, exosome/extracellular-vesicle RNA studies, endocrine-signalling studies, environmental-exposure studies and translational biomarker studies. Mechanistic support was categorised descriptively according to study type, biological relevance to ISS, mechanistic specificity, consistency with related findings and translational applicability. Original clinical, mechanistic and translational studies were treated as core evidence, whereas reviews and broader background articles were used only for contextual framing.

To further clarify the source-selection process, Supplementary Figure S1 presents a concise decision tree summarising how sources were identified, prioritised and incorporated into the structured narrative synthesis. The diagram illustrates the progression from database and reference-list searching to relevance-based source prioritisation, evidence-role assignment and extraction of mechanistic read-outs. It also distinguishes core evidence, including original mechanistic and translational studies, from contextual background sources, while making clear that case reports without mechanistic data and conference abstracts were not used as core evidence. This visual framework improves transparency without implying that the review followed a formal systematic-review or scoping-review protocol.

We prioritised original mechanistic and translational research reporting growth-plate phenotypes in human subjects, exosomal profiling, chondrocyte cultures, animal models or omics analyses. For each selected study, we extracted the disorder context, experimental model (human plasma or tissue, chondrocyte culture, rodent model or organoid) and key read-outs such as proliferation, hypertrophy, ECM maturation and ossification. Variables such as bone length, growth-plate thickness and expression of COL2A1, COL10A1, RUNX2, osteocalcin, osteopontin, alkaline phosphatase and matrix mineralisation were recorded. These read-outs were selected because they corresponded to the main biological processes used to organise the narrative synthesis: proliferation, hypertrophy, ECM maturation, mineralisation and ossification.

Evidence items were categorised into route classes—exosomal RNA, ECM defect, GH–IGF variation, environmental/inflammatory exposure and local signalling nodes—and the primary target process of each (proliferation, hypertrophy, ECM synthesis or ossification) noted. The level of mechanistic support was categorised descriptively as association, functional assay, rescue experiment or *in vivo* validation; this categorisation was not intended as formal certainty grading or risk-of-bias assessment. Data were synthesised narratively and organised across figures and tables using conceptual frameworks presenting growth-plate state-transition and the ECM–exosome interface. Exosomal RNA evidence was interpreted using current extracellular vesicle reporting principles, especially the need to define EV isolation, characterisation, cargo validation and functional uptake assays (Théry et al., 2018; Welsh et al., 2024).

To identify research gaps, we searched for under-represented intersections between ECM and exosomal biology. Conceptual gap mapping indicated strong support for growth-plate biology, moderate support for ECM and exosomal mechanisms and scarce evidence for their direct interaction. These gaps informed proposed experiments and directions for future research. This procedure should be understood as conceptual gap identification within a structured narrative synthesis, not as formal scoping-review evidence mapping. Gap identification focused on whether retrieved studies directly connected ECM features with extracellular-vesicle cargo, vesicle uptake or RNA-mediated signalling in growth-plate-related models.

## 3 Synthesis of evidence

### 3.1 Growth-plate chondrocyte state-transition failure in ISS

**FIGURE 1:**
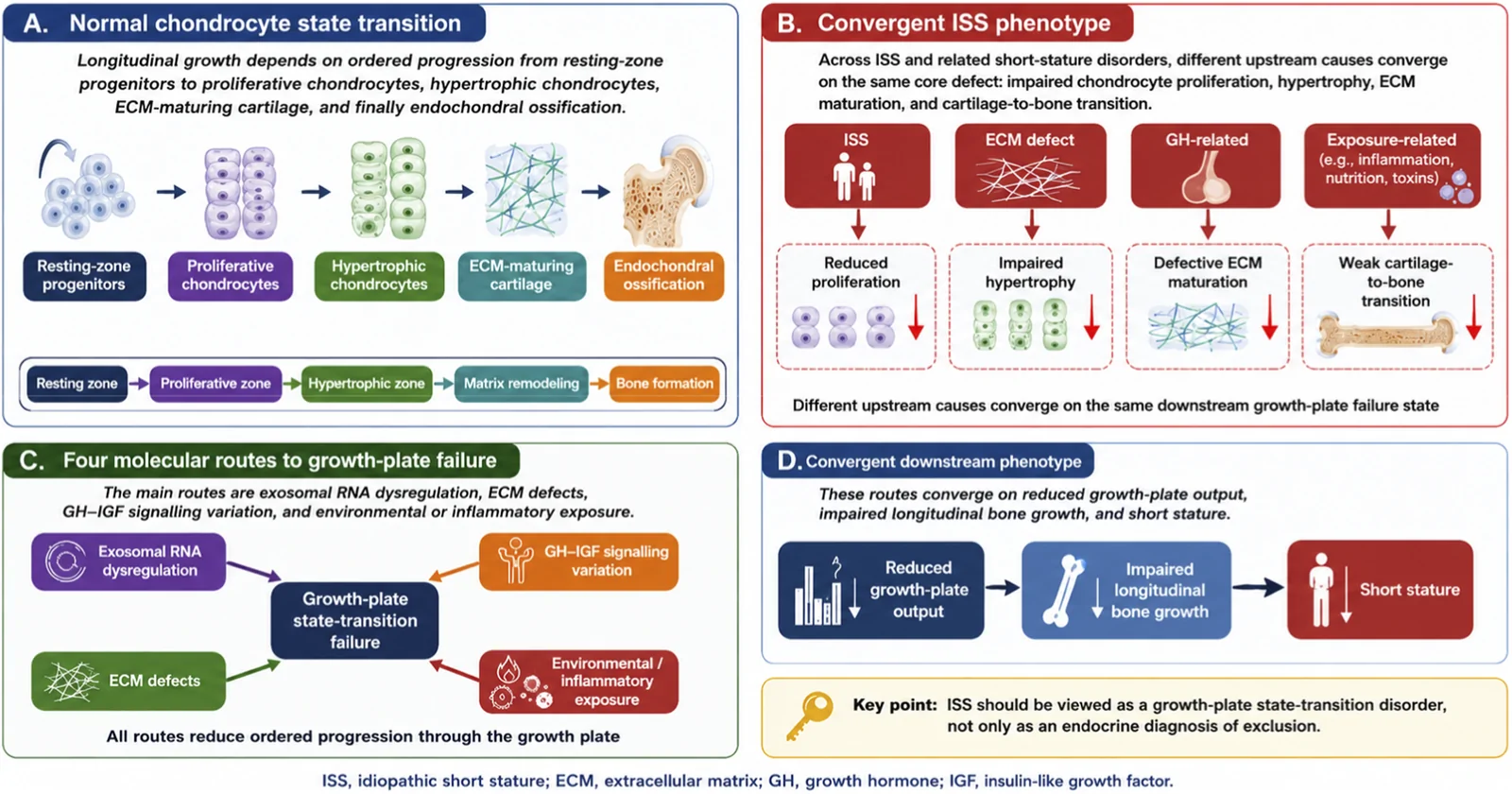
A convergent growth-plate failure model for idiopathic short stature **(A)** Normal chondrocyte state transition. Longitudinal growth depends on ordered progression from resting-zone progenitors to proliferative chondrocytes, hypertrophic chondrocytes, ECM-maturing cartilage, and finally endochondral ossification. **(B)** Convergent ISS phenotype. Across ISS and related short-stature disorders, different upstream is associated with converge on the same core defect: impaired chondrocyte proliferation, hypertrophy, ECM maturation, and cartilage-to-bone transition. **(C)** Four molecular route to growth-plate failure. The main routes are exosomal RNA dysregulation, ECM-defect entries, GH–IGF signalling variation, and environmental or inflammatory exposure. **(D)** Convergent downstream phenotype. These routes converge on reduced growth-plate output, impaired longitudinal bone growth, and short stature.

Longitudinal bone growth depends on coordinated transitions of growth-plate chondrocytes from resting-zone progenitors to proliferative, hypertrophic and matrix-mineralising states (Figure 1A) (Chen et al., 2023; Hallett et al., 2019). Across ISS and related growth disorders, the reviewed evidence indicates a recurring pattern of reduced chondrocyte proliferation, blunted hypertrophy, delayed ECM maturation and impaired cartilage-to-bone transition (Figure 1B) (Hallett et al., 2021; Tsang et al., 2015). These abnormalities appear to arise through multiple upstream routes, including dysregulated exosomal RNA, structural ECM-defect entries, altered GH–IGF signalling and environmental/inflammatory exposures (Figure 1C). Despite their mechanistic diversity, these routes converge on reduced growth-plate output and impaired longitudinal bone growth (Figure 1D).

Table 1 summarises these reported observations, which are supported by Supplementary Table S1. The evidence base included 46 unique studies; because individual studies could contribute to more than one evidence category, the coded category-level contributions were 17 ISS-related entries, 25 ECM-related defect entries, 18 GH-related disorder entries, 11 related short-stature disorder entries, and 10 related growth-plate disorder entries. Across 686 coded observations, most findings were descriptive/unclear (n = 497), while directional findings showed decreased/impaired outcomes (n = 80), increased/improved outcomes (n = 76), and mixed/dysregulated outcomes (n = 33). Across the ISS-related entries with directional read-outs, the dominant processes affected were chondrocyte hypertrophy and ossification; key markers such as RUNX2, osteocalcin and alkaline phosphatase were decreased, and the included models reported shortened bone length or impaired matrix mineralisation, supporting the interpretation that many ISS-associated models show impaired endochondral ossification (Liu et al., 2023; Wang et al., 2025). Across ECM-defect entries, loss of aggrecan or type II/X collagen was associated with shortened growth plates and reduced mineralisation in mice, rats and human cartilage; matrix architecture emerged as the important element of growth-plate maturation (Domowicz et al., 2009; Lauing et al., 2014; Taieb et al., 2023). GH–IGF-related entries showed variable effects on proliferation but consistent dysregulation of hypertrophy and ossification, while entries on ISS and growth-plate disorders highlighted that diverse conditions ultimately share reduced mineralisation and matrix-dependent maturation (Ono et al., 2013; Pellicciari et al., 2025). Overall, the synthesised evidence indicates that impaired proliferative-hypertrophic transition and matrix maturation are unifying features of ISS and related conditions, as summarised in Table 1 (Chen et al., 2022; Plachy et al., 2021).

**TABLE 1:** Key biological outcomes and parameters across ISS and related short-stature studies.

Context	Available reported numbers	Models represented	Dominant growth-plate process	Overall direction	Key parameters/read-outs	Interpretation
Idiopathic short stature (ISS)	17 studies; 135 observations: ↓30, ↑14, mixed 7, unclear 84; 102 read-outs	Human/child samples; exosomes; growth plate tissue; chondrocyte culture; rat model	Proliferation-hypertrophy-ossification axis; ECM maturation in a subset	Predominantly decreased or dysregulated	RUNX2, OCN, OPN, COL10A1, ALP, mineralisation, bone length/growth phenotype	ISS converges on impaired chondrocyte maturation and endochondral ossification
ECM-related defects	25 studies; 218 observations: ↓21, ↑24, mixed 9, unclear 164; 130 read-outs	Growth plate tissue; chondrocyte culture; mouse/rat models; human samples	ECM maturation coupled to hypertrophy and ossification	Mostly altered; decreased matrix/length in functional models	Proteoglycan/matrix, COL2A1, COL10A1, RUNX2, mineralisation, bone/body length	Matrix architecture is an important element of growth-plate maturation and linear growth
GH–IGF variation	18 studies; 186 observations: ↓13, ↑21, mixed 6, unclear 146; 77 read-outs	Chondrocyte culture; growth plate tissue; rodent models; human/child samples	Hypertrophy and ossification, with proliferation in many models	Variable; generally dysregulated growth response	Growth phenotype, COL2A1, COL10A1, bone length, mineralisation, RUNX2/OCN	GH–IGF variation modulates growth through chondrocyte expansion and maturation
Related short-stature disorders	11 studies; 62 observations: ↓11, ↑6, mixed 6, unclear 39; 63 read-outs	Chondrocyte culture; growth plate tissue; mouse model	Proliferation and ossification; hypertrophy in a subset	Mostly decreased or dysregulated	Growth phenotype, bone length, COL2A1, proteoglycan/matrix, OCN	Short-stature syndromes share reduced proliferative and matrix-dependent maturation
Related growth-plate disorders	10 studies; 85 observations: ↓5, ↑11, mixed 5, unclear 64; 50 read-outs	Chondrocyte culture; growth plate tissue; rodent models; human/child samples	Ossification-dominant phenotype with hypertrophy/proliferation changes	Mixed or dysregulated	Mineralization, growth phenotype, COL2A1/COL10A1, body length, bone length	Distinct growth-plate disorders converge on impaired mineralisation and endochondral progression

Abbreviations: ALP, alkaline phosphatase; COL2A1/COL10A1, collagen type II/X alpha 1 chain; ECM, extracellular matrix; GH, growth hormone; ISS, idiopathic short stature; OCN, osteocalcin; OPN, osteopontin; RUNX2, runt-related transcription factor-2. The direction column summarises the dominant pattern in the extracted rows; descriptive or mixed rows are reported as dysregulated.

### 3.2 Convergent effects of exosomal RNA, ECM, GH–IGF and environmental pathways on growth-plate failure

**FIGURE 2:**
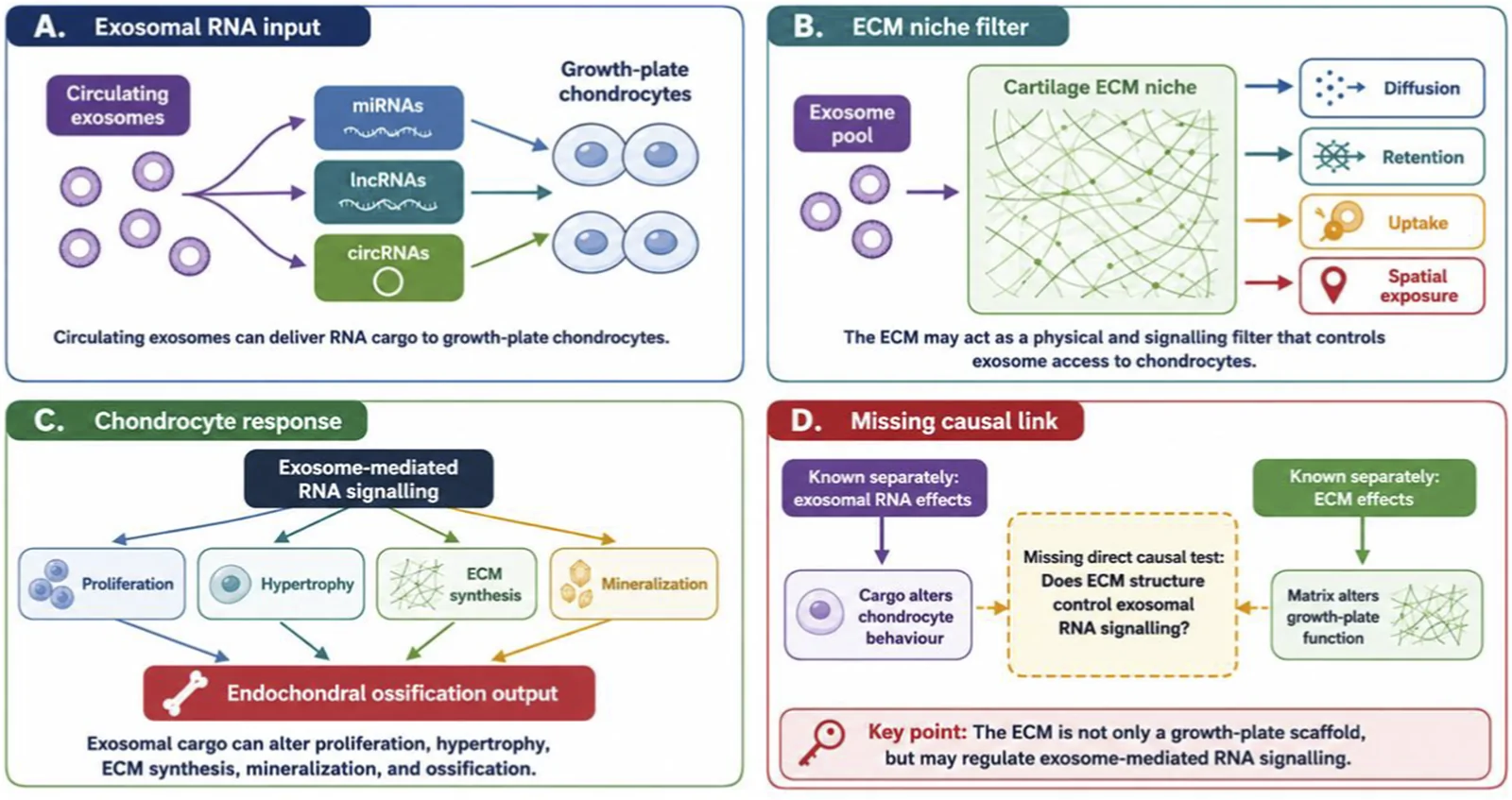
The proposed ECM–exosome interface influencing chondrocyte fate. **(A)** Exosomal RNA input. Circulating exosomes deliver miRNAs, lncRNAs, and circRNAs to growth-plate chondrocytes. **(B)** ECM niche filter. The cartilage ECM may regulate exosome access by controlling vesicle diffusion, retention, uptake, and spatial exposure to chondrocytes. **(C)** Chondrocyte response. Exosomal cargo can alter proliferation, hypertrophy, ECM synthesis, mineralisation, and endochondral ossification. **(D)** Missing causal link. Current studies show exosomal RNA effects and ECM effects separately, but few directly test whether ECM structure controls exosomal RNA signalling.

The reviewed evidence supports an emerging ECM–exosome interface in which EV may influence growth-plate chondrocyte behaviour through delivery of regulatory microRNAs, lncRNAs and circRNAs (Figure 2A) (Dai et al., 2020; Li et al., 2024). Broader cartilage and matrix-biology evidence suggests that the ECM may act not only as a structural scaffold but also as a regulatory microenvironment capable of modifying cell signalling and vesicle–cell interactions (Figure 2B) (Piccinini and Midwood, 2014). Studies in cartilage-lineage cells show that EV cargo can alter proliferation, hypertrophy, ECM homeostasis, mineralisation and ossification (Figure 2C). However, direct evidence linking cartilage ECM architecture to exosomal RNA uptake or activity in ISS remains limited, making this interface a plausible but incompletely validated mechanism (Figure 2D).

Supplementary Table S2 collates study-level molecular evidence supporting these routes, while Table 2 summarises the route-level interpretation. In the study-level molecular evidence, exosomal-RNA entries indicate that miR-26b-3p, ISS-related lncRNAs and circRNA_0079201 are variably up- or downregulated across human plasma, chondrocyte cultures and animal models. Despite heterogeneous cargo, all exosomal routes converge on defective proliferation, ECM synthesis and ossification; rescue experiments using engineered exosomes or RNA-targeted exosomal interventions partially restored chondrocyte proliferation and mineralisation (Liu et al., 2023; Yuan et al., 2024). Twenty ECM-defect studies show that mutations in ACAN, COL2A1 and COL10A1 impair matrix synthesis, hypertrophy and bone elongation (Chen et al., 2022; Lauing et al., 2014; Plachy et al., 2021). GH–IGF variation entries suggest that altered SMAD2/MAPK signalling modifies endocrine responsiveness but still results in growth-plate failure (Ono et al., 2013; Pellicciari et al., 2025). Environmental exposures entries and local signalling entries likewise produce phenotypes of reduced proliferation, abnormal hypertrophy and incomplete ossification (Shin et al., 2026; Wang et al., 2025). Thus, the synthesised molecular evidence indicates that diverse upstream mechanisms are associated with overlapping downstream processes, with Table 2 summarising the supporting studies.

**TABLE 2:** Divergent molecular routes converging on growth-plate failure.

Molecular route	Representative molecules	Primary target process	Evidence type	Degree of causality	Interpretation
Exosomal RNA	miR-26b-3p; ISSRL; circRNA_0079201	Proliferation; ECM synthesis; ossification; hypertrophy	Human sample; chondrocyte culture; omics; animal model; therapeutic rescue	Rescue experiment + *in vivo* validation	Available evidence suggests that exosomal RNA dysregulation involves heterogeneous cargo profiles but may converge on shared growth-plate outcomes, including altered proliferation, ECM synthesis, hypertrophic differentiation, and ossification. This interpretation is supported by findings from human samples, chondrocyte culture, omics analyses, animal models, and rescue-oriented studies, although direct cargo–target validation remains an important future step
ECM defect	ACAN; col2a1; col10a1; IGF-1; FGFR; FGFR1	ECM synthesis; ossification; proliferation; hypertrophy	Human sample; chondrocyte culture; omics; animal model; therapeutic rescue	Rescue experiment + *in vivo* validation	ECM-related abnormalities appear to represent a central route through which diverse genetic, structural, or matrix-associated defects may impair growth-plate function. Current evidence from human samples, chondrocyte culture, omics analyses, and animal models supports links between matrix disruption and altered ECM synthesis, proliferation, hypertrophic differentiation, and ossification
GH–IGF variation	IGF-1; SMAD2; MAPK1; MAPK3; SOX9	Growth-plate failure; proliferation; hypertrophy; ossification	Human sample; omics; chondrocyte culture; animal model; therapeutic rescue	Functional test + *in vivo* validation	GH–IGF pathway variation may modify endocrine responsiveness while still converging on impaired growth-plate activity, including altered proliferation, hypertrophic differentiation, and ossification. Evidence from human samples, omics analyses, chondrocyte culture, animal models, and rescue-oriented studies supports this interpretation, but its predictive value for treatment response requires further prospective validation
Environmental exposure	​	Proliferation; ossification; hypertrophy	Chondrocyte culture; animal model; omics; human sample	Functional test + *in vivo* validation	Exposure-associated perturbations appear mechanistically distinct from inherited or endocrine pathways, but they may produce overlapping growth-plate phenotypes, including altered proliferation, hypertrophic differentiation, and ossification. Evidence from chondrocyte culture, animal models, omics analyses, and human samples suggests that non-genetic inputs can contribute to ISS-like growth-plate dysfunction, although stronger cohort-level validation is needed
Local signalling	FGFR; FGFR2; FGFR4; SMAD2; SMAD4; mapk	Ossification; growth-plate failure; proliferation; hypertrophy	Human sample; chondrocyte culture; animal model; omics; therapeutic rescue	Rescue experiment + *in vivo* validation	Altered local signalling pathways appear to provide another convergent mechanism linking distinct upstream molecular defects to growth-plate dysfunction. Evidence from human samples, chondrocyte culture, animal models, omics analyses, and rescue experiments supports associations with impaired proliferation, hypertrophic differentiation, and ossification, while future studies should clarify pathway hierarchy and causal interactions

This table is intentionally route-level and summarises why upstream evidence differs across studies while downstream growth-plate failure remains shared. Table 2 compares the divergent upstream mechanisms that can produce similar growth-plate failure. The evidence indicates that ISS-like phenotypes may arise through exosomal RNA dysregulation, ECM-defect entries, GH–IGF, signalling variation, environmental/inflammatory exposures, or altered local signalling. The table distinguishes association from functional and rescue-level evidence, highlighting the molecular heterogeneity hidden within the ISS label.

### 3.3 Strength of evidence for growth-plate failure and ECM–exosome coupling

The integrated model raises the possibility that the cartilage matrix may do more than provide structural support and could influence vesicular access to chondrocytes, with potential downstream effects on RNA signalling. This statement is interpreted as a hypothesis-generating model rather than a demonstrated mechanism in ISS. At present, direct evidence supports exosome-mediated regulation of chondrocyte proliferation, hypertrophy and ossification in ISS, but not direct ECM-dependent control of exosome uptake or RNA activity in human growth-plate chondrocytes (Liu et al., 2023; Wang et al., 2025). Available evidence indicates that exosomal cargo can reprogramme chondrocyte behaviour, yet no study has directly tested whether ECM composition controls exosome uptake (Figure 2). Therefore, throughout this review, the term “ECM–exosome growth-plate axis” is used as a conceptual framework to organise emerging evidence, not as proof of a validated causal pathway in ISS. The evidence base supports growth-plate biology, chondrocyte proliferation, hypertrophy and endochondral ossification more strongly than it supports a direct ECM–exosome interface in ISS (Supplementary Figure S2A) (Chen et al., 2023; Hallett et al., 2019). To describe the level of mechanistic support, each of the 46 selected studies was assigned descriptively to the highest applicable support category: association-level evidence (n = 8), functional assay (n = 2), rescue experiment (n = 3) and *in vivo* validation (n = 25). Conceptual gap mapping showed that lower-tier associative evidence was concentrated in genetic, endocrine-response and contextual biomarker literature, whereas in vivo-validation evidence was concentrated in growth-plate, ECM-defect and local-signalling models.

In contrast, exosomal RNA mechanisms and ECM-defect entries have moderate support, but these two areas are usually studied as separate research streams rather than as an integrated signalling system (Supplementary Figure S2B) (Dai et al., 2020; Taieb et al., 2023). The weakest evidence area is the direct causal interface between ECM structure and exosome-mediated RNA signalling in ISS, with no mapped source evidence directly testing whether ECM composition, stiffness or matrix organisation controls exosome uptake and RNA activity in growth-plate chondrocytes (Supplementary Figure S2C) (Piccinini and Midwood, 2014; Tiffany and Harley, 2022). Thus, the evidence-based conclusion is that ECM abnormalities and exosomal RNA dysregulation can each affect growth-plate-relevant processes, whereas their bidirectional interaction remains an inferred and testable hypothesis. Future studies should directly test whether defined ECM properties, including collagen composition, proteoglycan content, stiffness and matrix organisation, alter exosome binding, uptake, cargo release and downstream RNA signalling in growth-plate chondrocytes (Liu et al., 2023). Such experiments are required before the ECM–exosome axis can be described as an established mechanism in ISS.

### 3.4 Knowledge gaps in ECM–exosome signalling and ISS

**FIGURE 3:**
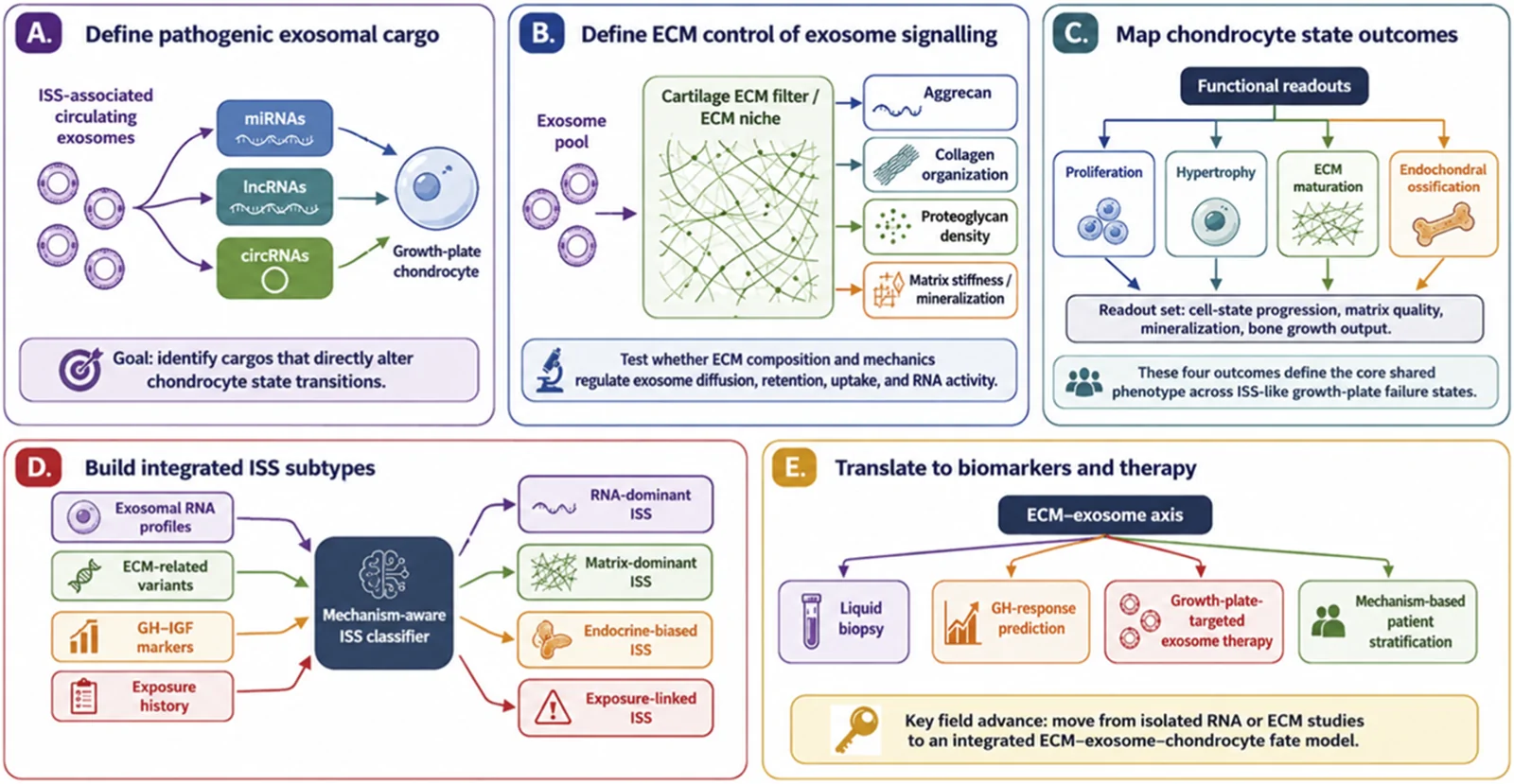
Research roadmap for validating the ECM–exosome growth-plate axis. **(A)** Define pathogenic exosomal cargo. Identify ISS-associated exosomal miRNAs, lncRNAs, and circRNAs that alter chondrocyte state transitions. **(B)** Define ECM control of exosome signalling. Test whether aggrecan, collagen organisation, proteoglycan density, matrix stiffness, and mineralisation regulate exosome uptake and RNA activity. **(C)** Map chondrocyte state outcomes. Measure proliferation, hypertrophy, ECM maturation, and endochondral ossification as the core functional read-outs. **(D)** Build integrated ISS subtypes. Combine exosomal RNA profiles, ECM-related variants, GH–IGF markers, and exposure history to classify ISS by mechanism. **(E)** Translate to biomarkers and therapy. Use the axis to guide liquid biopsy, GH-response prediction, and growth-plate-targeted exosome therapy.

Several knowledge gaps remain before the ECM–exosome growth-plate axis can be considered a validated mechanism in ISS. First, pathogenic exosomal cargo must be defined by identifying ISS-associated miRNAs, lncRNAs and circRNAs that alter chondrocyte transitions (Figure 3A) (Nakamichi et al., 2020; Thakore and Delany, 2025). Second, future studies should test whether ECM parameters, including aggrecan content, collagen organisation, proteoglycan density, stiffness and mineralisation, regulate exosome uptake and RNA activity (Figure 3B). Third, proliferative, hypertrophic and ossification outcomes should be mapped as core read-outs across experimental models (Figure 3C). Fourth, integrated ISS subtypes may be developed by combining exosomal profiles, ECM-related variants, GH–IGF markers and exposure history (Figure 3D). Finally, these findings may support future biomarker development, GH-response prediction and targeted exosome-based therapeutic strategies (Figure 3E) (Lv et al., 2025; Pellicciari et al., 2025).

Table 3 translates these unresolved questions into proposed experimental approaches. It identifies five gaps: (1) whether the cartilage ECM directly controls exosome uptake and cargo release; (2) which exosomal RNAs regulate ECM assembly and hypertrophic transition; (3) the tissue origins of ISS-associated exosomes; (4) whether proposed mechanisms operate in human growth-plate cells; and (5) whether ECM–exosome signatures predict GH responsiveness. The proposed approaches include comparing fluorescently bar-coded exosomes in ECM-normal versus ECM-disrupted cartilage, gain- and loss-of-function exosomal RNA manipulations with luciferase target validation, tissue-resolved exosome profiling in paediatric cohorts, applying perturbations in human growth-plate organoids, and prospective studies combining exosomal and ECM markers with growth outcomes. Each experiment lists minimum read-outs (proliferation, hypertrophy, ECM maturation, uptake, RNA activity, ossification) and suggests appropriate models (human chondrocytes, organoids, hPSC-derived cartilage, animal models, clinical cohorts). Collectively, these proposed approaches define a research agenda to test whether the currently correlative ECM–exosome relationship reflects a causal, clinically relevant mechanism (Tam et al., 2021; Xiong et al., 2025; Zhang et al., 2025).

**TABLE 3:** Key research gaps and proposed experiments for the ECM–exosome growth-plate axis.

Research gap	Why it matters	Decisive experiment	Minimum read-outs	Best model	Expected advance
Does the cartilage ECM niche directly control exosome uptake and intracellular cargo release in growth-plate chondrocytes?	This is the central unresolved mechanistic question for the ECM–exosome axis. Without direct uptake evidence and cargo-release evidence, it remains unclear whether ECM changes regulate exosomal RNA activity or are parallel correlates of growth-plate dysfunction	Compare fluorescently barcoded native and engineered ISS/control exosomes in ECM-normal versus ECM-disrupted 3D cartilage systems, then quantify binding, internalization, endosomal escape, and pathway activation after ECM perturbation or rescue	Exosome uptake; intracellular RNA activity; proliferation; hypertrophy; ECM maturation; ossification	Human chondrocytes + growth-plate organoid	Would help determine whether ECM state influences exosomal signalling and would provide experimental evidence needed to move beyond a purely correlative model. This remains a priority because current evidence appears insufficient to demonstrate ECM-dependent exosome uptake and intracellular cargo activity directly
Which exosomal RNAs directly regulate ECM assembly, matrix maturation, and hypertrophic transition in growth-plate chondrocytes?	Existing studies suggest associations between exosomal cargo and cellular phenotype, but direct evidence that specific cargo alters ECM production remains limited. Clarifying this distinction would help explain whether different upstream signals converge on shared defects in cartilage matrix maturation	Use gain/loss-of-function exosomes carrying candidate ISS RNAs in chondrocyte or organoid systems, combined with luciferase target validation and rescue experiments that restore ECM composition or block the predicted cargo–target interaction	RNA activity; ECM maturation; COL2A1/COL10A1; proliferation; hypertrophy; mineralisation/ossification	Human chondrocytes + hPSC-derived cartilage model	Could identify candidate exosomal cargo–target pairs linking RNA dysregulation to altered matrix maturation and help prioritize targets for further validation. This remains an important next step because current evidence does not yet define which exosomal RNAs directly regulate ECM assembly or hypertrophic transition
What tissues generate the exosomes associated with ISS, and which cellular source is most relevant to growth-plate dysfunction?	Circulating exosomes likely arise from multiple tissues and cell types. Without tissue-of-origin resolution, it is difficult to determine whether plasma exosome signals reflect growth-plate biology or can support reliable patient stratification	Perform tissue-resolved exosome profiling with matched plasma and source-tissue signatures, then test whether exosomes from candidate tissues reproduce growth-plate phenotypes in recipient chondrocytes and in vivo models	Exosome uptake; RNA cargo composition; proliferation; hypertrophy; ECM maturation; ossification	Paediatric cohort + animal model	Could identify candidate tissue sources contributing to ISS-associated exosome signatures and help connect biomarker observations with tissue-level mechanisms. This remains unresolved because circulating exosome findings do not by themselves establish the tissue source or growth-plate relevance of the signal
Do the proposed ECM–exosome mechanisms operate in human growth-plate cells rather than only in surrogate cell lines or animal cartilage?	Human translational relevance remains a major barrier to establish causality. Mechanisms observed only in surrogate systems require validation in human growth-plate-relevant models before they can inform precision ISS models or intervention studies	Test prioritized ECM and exosome perturbations in human growth-plate organoids or hPSC-derived chondrocyte systems, with matched comparison to primary human samples or paediatric cohort signatures where available	Proliferation; hypertrophy; ECM maturation; exosome uptake; RNA activity; ossification	Growth-plate organoid + hPSC model	Could provide human-model validation needed to assess whether findings from animal or surrogate systems are clinically relevant. This remains a priority because proposed ECM–exosome mechanisms require confirmation in human growth-plate-relevant cells or organoid systems
Can ECM–exosome signatures predict GH responsiveness and distinguish mechanistically different ISS subgroups before treatment?	ISS is clinically heterogeneous. A validated predictive framework could help translate pathway heterogeneity into patient stratification and reduce on empirical treatment selection	Build a prospective baseline-to-response study combining exosome cargo, ECM-related markers, and pathway-state measurements, then test whether these features predict growth velocity and height gain after GH or rescue-oriented intervention	RNA activity; exosome uptake proxy/cargo status; ECM maturation markers; proliferation/hypertrophy state; bone growth outcomes	Paediatric cohort	Could test whether ECM–exosome signatures are associated with GH responsiveness and whether they can distinguish clinically relevant ISS subgroups. This remains a future direction because prospective evidence is needed to determine whether ECM–exosome markers predict growth outcomes after GH treatment

This table focuses on proposed next-step experiments rather than summarizing existing figures. Table 3 outlines experimental approaches that could help validate the ECM–exosome growth-plate axis in ISS. A central unresolved question is whether the cartilage ECM, niche regulates exosomal RNA, delivery and activity in growth-plate chondrocytes. Priority future experiments should test ECM-dependent exosome uptake, exosomal regulation of ECM, maturation, tissue origin of ISS, exosomes, human growth-plate relevance, and prediction of GH, response.

### 3.5 Downstream phenotypes across ISS, ECM-defect and GH-related studies

Supplementary Table S1 synthesises study-level evidence across ISS, ECM-defect, GH-related and related short-stature contexts. Across these categories, the most consistent downstream processes were impaired chondrocyte proliferation, hypertrophy, ECM maturation and ossification. The most frequently recurring read-outs included RUNX2, COL10A1, COL2A1, OCN, OPN, ALP, mineralisation, proteoglycan/matrix measures, bone length and general growth-phenotype endpoints. Rather than identifying a single molecular defect common to all studies, the table shows convergence on a limited set of growth-plate outputs across diverse upstream mechanisms (Chen et al., 2018; Ma et al., 2020).

Several exceptions and sources of heterogeneity were also evident. Some studies reported increased, mixed or descriptive changes rather than uniformly decreased read-outs, and a small number of rescue experiments suggested partial reversibility of growth-plate phenotypes. GH-related studies more often reported systemic growth outcomes, such as height SDS or growth response, whereas ECM-defect studies more consistently captured matrix architecture, hypertrophy and ossification read-outs. Overall, Supplementary Table S1 supports a cross-cutting pattern in which diverse ISS, ECM-defect and GH–IGF variation converge on disrupted chondrocyte state transitions and impaired cartilage-to-bone progression.

### 3.6 Molecular and phenotypic read-outs of the ECM–exosome axis

Supplementary Table S2 groups molecular and pathway-level evidence into exosomal RNA, ECM-defect, GH–IGF and local-signalling clusters. The descriptive mechanistic-support category assigned to each study is reported in Supplementary Table S1, and the molecular-route conceptual-gap summary is shown in Supplementary Table S2. Across these clusters, exosomal RNA studies provided the most detailed functional evidence for altered proliferation and ossification, particularly where *in vivo* models, functional assays or rescue experiments were included. However, the table also shows that direct cargo-to-target validation and causal rescue remain inconsistent, limiting the strength of inference for specific RNA-mediated mechanisms. ECM-related clusters were most closely associated with matrix maturation, hypertrophy and ossification, but matrix-rescue experiments and direct tests linking matrix defects to growth-plate phenotypes were limited. GH–IGF-related evidence was comparatively sparse and focused mainly on growth-plate phenotype or treatment responsiveness rather than direct ECM–exosome mechanisms. Local-signalling clusters, including osteochondral, SMAD/BMP, FGF/FGFR, MAPK/ERK, IHH–PTHrP and Wnt-related pathways, appeared across multiple phenotype categories, but most entries remained associative rather than pathway-causal. Taken together, Supplementary Table S2 indicates that the strongest cross-cutting evidence concerns convergence on proliferation, hypertrophy, ECM maturation and ossification, whereas direct bidirectional coupling between ECM properties and exosomal RNA signalling remains insufficiently tested (Wang et al., 2025; Wu et al., 2022).

## 4 Discussion

Our synthesis identifies a convergent downstream pattern across mechanistically diverse ISS studies: impaired chondrocyte proliferation, delayed hypertrophy, disrupted ECM maturation and reduced endochondral ossification. This pattern was observed across exosomal RNA, ECM-defect, GH–IGF and environmental/inflammatory exposure streams, suggesting that heterogeneous upstream perturbations may converge on an overlapping growth-plate output. We therefore avoid interpreting the proposed axis as a proven disease mechanism and instead use it to organise existing evidence and define experimentally testable predictions. ISS is increasingly recognised as a disorder of growth-plate biology rather than a purely endocrine diagnosis. Our synthesis supports the interpretation that disparate upstream perturbations—exosomal RNA dysregulation, ECM-defect entries, GH–IGF variation, and environmental and inflammatory exposures—are associated with an overlapping pattern of impaired chondrocyte proliferation, hypertrophy, ECM maturation and endochondral ossification. This convergence is consistent with overlapping downstream processes in cartilage-to-bone transition; however, whether ECM–exosome interactions mediate these processes in ISS remains to be directly tested (Liu et al., 2023; Yuan et al., 2024).

Clinically, this framework may help reframe ISS as a heterogeneous diagnostic category rather than a single unexplained growth phenotype. For paediatricians and clinical geneticists, the practical implication is not that ECM–exosome testing is ready for routine care, but that ISS patients could be stratified according to dominant evidence streams: suspected growth-plate/ECM-defect entries, GH–IGF-axis variation, environmental or inflammatory exposure history, and emerging extracellular-vesicle/RNA biomarkers. In current practice, this supports a stepwise diagnostic workup that combines auxology, growth velocity, bone age, GH–IGF assessment, dysmorphology or skeletal-disproportion screening, targeted genetic testing or exome sequencing when indicated, and careful exposure history. ECM–exosome profiling should presently be regarded as a research-layer classification tool rather than a validated clinical diagnostic test.

We therefore adopted a structured narrative-review framework rather than a systematic or scoping-review framework. Study selection was therefore relevance-based and iterative, with priority given to sources that informed potential mechanistic relationships among ISS, growth-plate regulation, ECM maturation and exosome-mediated RNA signalling. Growth-plate terms were included to capture studies addressing chondrocyte proliferation, hypertrophy, ECM remodelling and endochondral ossification as processes relevant to the review question.

### 4.1 Current state of knowledge

The strongest evidence base comes from studies of growth-plate biology and related growth disorders, where multiple human and animal models consistently show reduced COL2A1/COL10A1 expression, decreased RUNX2 and osteocalcin, diminished alkaline phosphatase activity and shortened bone length across ISS and related disorders (Mizuhashi et al., 2018; Newton et al., 2019; Song et al., 2025). Moderate evidence supports separate roles for exosomal RNAs and ECM-defect entries: circulating exosomes can deliver miRNAs, lncRNAs and circRNAs to chondrocytes and alter proliferation, ECM synthesis and mineralisation in selected models, while mutations in aggrecan, type II/10 collagen, FGFR or SMAD pathways can disrupt matrix architecture and hypertrophic differentiation (Kalluri and LeBleu, 2020; Marouli et al., 2017; van Niel et al., 2018; Yengo et al., 2022). This distinction is critical because parallel involvement of ECM defects and exosomal RNA changes does not by itself establish a functional ECM–exosome interaction. Supplementary evidence density mapping highlights this gap: growth-plate biology is well represented but direct ECM–exosome interplay remains under-studied (Huleihel et al., 2016; Humphrey et al., 2014; Hynes, 2009).

### 4.2 Controversies and contradictions

Individual exosomal cargo studies report both increased and decreased miR-26b-3p, miR-185-3p, miR-17-3p, circRNA_0079201 and other RNAs, yet downstream phenotypes often involve reduced proliferation, delayed hypertrophy and poor ossification (Liu et al., 2023; Wang et al., 2025; Wu et al., 2022). Similarly, GH-IGF and local signalling variations (e.g., altered SMAD/MAPK/Wnt7b) show variable endocrine responsiveness and are associated with phenotypes consistent with growth-plate dysfunction (Decker et al., 2017; Lui et al., 2018; Oichi et al., 2020). Such heterogeneity underscores that ISS is mechanistically diverse, but the downstream processes converge on a limited set of chondrocyte state transitions. Recognizing this convergence may be useful for future diagnostics: it suggests that ISS subtypes might be stratified by upstream route and may share downstream processes that could become therapeutic targets if validated (Andrade et al., 2022; Marouli et al., 2017; Yengo et al., 2022).

### 4.3 Critical appraisal

Most exosome studies rely on animal models or surrogate cell lines, with limited human growth-plate validation. Exosome isolation methods vary, and many studies measure circulating exosomes without resolving their tissue of origin (Kalluri and LeBleu, 2020; van Niel et al., 2018). ECM studies often manipulate single matrix components or signalling nodes, which may not recapitulate the complex, multiscale properties of cartilage. Few experiments test whether exosomal RNAs directly regulate matrix assembly, or whether ECM stiffness, proteoglycan density or mineralisation state modulates exosome uptake. In addition, descriptive mechanistic-support categorisation indicates that rescue experiments are limited and direct human growth-plate validation remains sparse. These limitations hinder causal inference and highlight the need for standardised models and read-outs (Engler et al., 2006; Discher et al., 2005; Humphrey et al., 2014). Human growth-plate organoids, hPSC-derived chondrocyte systems and engineered cartilage platforms would therefore be valuable for testing whether ECM-dependent exosome signalling operates in a human developmental context rather than only in simplified culture models (Tam et al., 2021; Xiong et al., 2025; Zhang et al., 2025).

### 4.4 Future directions

Consistent with Table 3, our analysis identifies five research priorities. First, test whether and how the cartilage ECM influences exosome uptake and intracellular cargo release—comparative uptake studies with engineered exosomes in ECM-normal versus ECM-defective organoids are needed (Huleihel et al., 2016; Xiong et al., 2025). Second, identify exosomal RNAs that directly regulate ECM synthesis and hypertrophic transition, using gain- and loss-of-function exosomes with target validation (Liu et al., 2023; Yuan et al., 2024). Third, establish the tissue sources of ISS-associated exosomes through matched plasma and tissue profiling (Kalluri and LeBleu, 2020; van Niel et al., 2018). Fourth, validate ECM–exosome mechanisms in human growth-plate organoids and hPSC-derived cartilage (Tam et al., 2021; Xiong et al., 2025). Fifth, evaluate whether combined ECM and exosome signatures predict GH responsiveness and enable mechanism-based ISS subclassification (Marouli et al., 2017; Yengo et al., 2022; Pellicciari et al., 2025). Addressing these gaps could help determine whether the ECM–exosome axis can move from a conceptual model toward a clinically actionable mechanism.

### 4.5 Proposed model/framework

If validated, elucidating the ECM–exosome growth-plate axis could help refine ISS management. However, these translational possibilities should be interpreted cautiously because most evidence remains preclinical, indirect or model-based, and no ECM–exosome-guided diagnostic algorithm or exosome-based therapy has yet been validated for routine management of ISS. Liquid biopsy of circulating exosomal RNAs and matrix-derived fragments could provide minimally invasive biomarkers for ISS subtype classification and treatment prediction (Kalluri and LeBleu, 2020; Shin et al., 2026). Engineering exosomes with therapeutic cargos to restore proliferation, promote ECM maturation or deliver matrix-stabilizing molecules may offer growth-plate-targeted interventions (Yuan et al., 2024; Lv et al., 2025). In the longer term, integrating exosome profiling, ECM genetics, endocrine markers and exposure history into clinical practice could move ISS from an umbrella diagnosis of exclusion to a stratified disorder with tailored therapies and prognostic guidance (Andrade et al., 2022; Toni et al., 2023). Ultimately, the proposed ECM–exosome framework may help reframe ISS as a potentially molecularly tractable disorder of chondrocyte state transition, while motivating further studies toward more precise treatment strategies.

## 5 Conclusion

Across figures and tables, a coherent picture emerges: ISS is viewed, at least in part, as a growth-plate state-transition disorder characterised by impaired proliferation, hypertrophy, ECM maturation and endochondral ossification. Diverse upstream perturbations—including exosomal RNA dysregulation, ECM-defect entries, GH–IGF variation, environmental/inflammatory exposures and altered local signalling—appear to be associated with similar downstream phenotypes in many studied models. This statement should be interpreted as a proposed mechanistic model, not as a conclusion directly demonstrated in ISS or human growth-plate tissue. Until such evidence is available, the ECM–exosome growth-plate axis should be regarded as a conceptual framework for future research rather than a validated causal pathway. This integrated approach could help refine ISS classification, improve GH-response prediction and inform future exploration of exosome-based growth-plate therapeutics.
