# Supplementary material for: Emerging molecular mechanisms of the ECM–exosome growth-plate axis in idiopathic short stature
Source: Front Cell Dev Biol. 2026 Aug 18;14:1898879. doi: 10.3389/fcell.2026.1898879 (PMC13530325; doi:10.3389/fcell.2026.1898879)
Supplement: Supplementary file 1 [file Table1.docx]

**Table S1. Consistent biological outcomes across ISS and related short-stature studies.**

| Source study | Disorder/context + theme | Models represented | Processes/readouts captured | Outcome direction | Key parameters/readouts | Combined interpretation |
| --- | --- | --- | --- | --- | --- | --- |
| (Yuan et al, 2024) | ISS Themes: Signalling; Growth plate | Outcome: Chondrocytes / cell culture; Exosomes; Growth plate tissue; Human / child samples; Rat model Inventory: Human; Animal; Chondrocyte culture; Omics | Outcome transitions: Proliferation; Hypertrophy; Ossification; ECM maturation Inventory readouts: Proliferation; Hypertrophy; Ossification | Decreased (n=5); Increased (n=3); Descriptive / unclear (n=7) | COL10A1; RUNX2; OCN; OPN; COL2A1; Growth phenotype readout | Integrates ISS outcome data with Signalling; Growth plate evidence domains; mapped biology centers on Proliferation; Hypertrophy; Ossification; ECM maturation. |
| (Wang et al, 2025) | ISS Themes: Exosome; Signalling; Growth plate | Outcome: Chondrocytes / cell culture; Exosomes; Growth plate tissue; Human / child samples; Rat model; Unclear Inventory: Animal; Chondrocyte culture; Engineered exosome; Human | Outcome transitions: Proliferation; Hypertrophy; ECM maturation; Ossification Inventory readouts: Proliferation; Hypertrophy; Ossification; Growth-plate phenotype | Decreased (n=5); Increased (n=1); Descriptive / unclear (n=13) | COL10A1; RUNX2; OCN; OPN; Growth phenotype readout; Growth-plate thickness | Integrates ISS outcome data with Exosome; Signalling; Growth plate evidence domains; mapped biology centers on Proliferation; Hypertrophy; ECM maturation; Ossification. |
| (Shin et al, 2026) | ISS Themes: Signalling; Growth plate | Outcome: Human / child samples; Unclear Inventory: Human; Omics; Animal | Outcome transitions: Proliferation; Hypertrophy; ECM maturation; Ossification Inventory readouts: Growth-plate phenotype | Decreased (n=1); Increased (n=2); Descriptive / unclear (n=1) | Phenotype endpoint not directly specified | Integrates ISS outcome data with Signalling; Growth plate evidence domains; mapped biology centers on Proliferation; Hypertrophy; ECM maturation; Ossification. |
| (Liu et al, 2020) | ISS; Related short-stature disorder Themes: Signalling; Growth plate | Outcome: Chondrocytes / cell culture; Human / child samples; Rat model; Unclear; Growth plate tissue Inventory: Human; Animal; Chondrocyte culture; NR | Outcome transitions: Proliferation; Hypertrophy; ECM maturation; Ossification Inventory readouts: Growth-plate phenotype; Proliferation; Ossification; Hypertrophy | Decreased (n=3); Increased (n=1); Mixed / bidirectional (n=3); Descriptive / unclear (n=8) | COL10A1; RUNX2; ALP; Mineralization; Growth phenotype readout; hsa_circRNA_0079201; miR-140-3p; SMAD2 | Integrates ISS and related short-stature evidence; mapped biology centers on circRNA_0079201-mediated suppression of chondrocyte proliferation, hypertrophy, and endochondral ossification. |
| (Wu et al, 2022) | ISS Themes: Signalling; Growth plate | Outcome: Chondrocytes / cell culture; Growth plate tissue; Human / child samples; Rat model; Unclear Inventory: Animal; Human; Omics; Chondrocyte culture | Outcome transitions: Proliferation; ECM maturation; Ossification; Hypertrophy; Growth-plate state transition not directly specified Inventory readouts: Growth-plate phenotype; Ossification; Proliferation; ECM maturation; Hypertrophy | Decreased (n=3); Mixed / bidirectional (n=1); Descriptive / unclear (n=10) | RUNX2; OCN; OPN; ALP; Bone length; Growth phenotype readout; hsa_circ_0008870; miR-185-3p; MAPK1 | Integrates ISS outcome data with exosome/signalling/growth-plate evidence domains; mapped biology centers on hsa_circ_0008870-mediated regulation of proliferation, hypertrophy, ECM maturation, and endochondral ossification. |
| (Liu et al, 2026) | ISS Themes: Growth plate | Outcome: Chondrocytes / cell culture Inventory: Human; Animal; Chondrocyte culture | Outcome transitions: Proliferation; Hypertrophy; ECM maturation; Ossification Inventory readouts: Proliferation; Ossification; Hypertrophy | Decreased (n=1); Descriptive / unclear (n=1) | COL10A1; RUNX2 | Integrates ISS outcome data with Growth plate evidence domains; mapped biology centers on Proliferation; Hypertrophy; ECM maturation; Ossification. |
| (Yuan et al, 2021) | ISS Themes: Growth plate; inflammation; biomarker | Outcome: Chondrocytes / cell culture; Human / child samples Inventory: Human; Animal; Omics; Chondrocyte culture | Outcome transitions: ECM maturation; Ossification Inventory readouts: Growth-plate phenotype; ECM maturation; Ossification | Descriptive / unclear (n=2) | RUNX2; OCN; OPN; Mineralization; C1QA; C1QB; collagen X; collagen II | Integrates ISS biomarker and growth-plate outcome data; mapped biology centers on complement/inflammatory markers and altered chondrocyte ossification-related readouts. |
| (Chen et al, 2024) | ISS; GH-related disorder Themes: Signalling | Outcome: Human / child samples; Unclear Inventory: Human; Animal; Omics | Outcome transitions: Proliferation; Hypertrophy; Ossification; Growth-plate state transition not directly specified Inventory readouts: Growth-plate phenotype | Descriptive / unclear (n=2) | Height SDS | Integrates ISS; GH-related disorder outcome data with Signalling evidence domains; mapped biology centers on Proliferation; Hypertrophy; Ossification; Growth-plate state transition not directly specified. |
| (Paparella et al, 2025) | ISS Themes: Signalling; genetic diagnosis; Growth plate | Outcome: Chondrocytes / cell culture; Growth plate tissue; Human / child samples Inventory: Human; Animal; Chondrocyte culture; Genetic/clinical review | Outcome transitions: Proliferation; Hypertrophy; ECM maturation; Growth-plate signalling Inventory readouts: Proliferation; Hypertrophy; ECM maturation; Growth-plate phenotype | Descriptive / unclear (n=2) | COL2A1; GH–IGF axis genes; ECM genes; growth-plate pathway genes; genetic testing | Integrates ISS genomic evidence with signalling and growth-plate domains; mapped biology centers on GH–IGF, ECM, and growth-plate pathway variants contributing to ISS. |
| (Xiong et al, 2025) | ISS; ECM defect; GH-related disorder; Related growth-plate disorder Themes: Signalling; Growth plate | Outcome: Chondrocytes / cell culture; Growth plate tissue; Human / child samples; Rat model; Unclear Inventory: Human; Chondrocyte culture; Animal; Omics | Outcome transitions: Proliferation; Hypertrophy; Ossification; ECM maturation Inventory readouts: Ossification; Hypertrophy; Proliferation | Decreased (n=1); Increased (n=2); Descriptive / unclear (n=14) | COL2A1; COL10A1; Mineralization; Proteoglycan / matrix | Integrates ISS; ECM defect; GH-related disorder; Related growth-plate disorder outcome data with Signalling; Growth plate evidence domains; mapped biology centers on Proliferation; Hypertrophy; Ossification; ECM maturation. |
| (Chen et al, 2025) | ISS; ECM defect Themes: Signalling; Growth plate | Outcome: Chondrocytes / cell culture; Growth plate tissue; Unclear Inventory: Animal; Chondrocyte culture; NR | Outcome transitions: Proliferation; Hypertrophy; ECM maturation; Ossification Inventory readouts: Ossification; Hypertrophy; Proliferation; Growth-plate phenotype; ECM maturation | Decreased (n=2); Increased (n=1); Descriptive / unclear (n=6) | Growth-plate thickness; RUNX2; ALP; COL2A1; Mineralization; Bone length; Proteoglycan / matrix | Integrates ISS; ECM defect outcome data with Signalling; Growth plate evidence domains; mapped biology centers on Proliferation; Hypertrophy; ECM maturation; Ossification. |
| (Thakore & Delany, 2025) | ISS; ECM defect Themes: Exosome; Signalling; miRNA regulation | Outcome: Chondrocytes / cell culture; Rat model; Growth-plate cartilage literature Inventory: Animal; Chondrocyte culture; Review | Outcome transitions: Proliferation; Hypertrophy; ECM maturation; Ossification Inventory readouts: Growth-plate phenotype; Ossification; Hypertrophy; Proliferation | Increased (n=2); Descriptive / unclear (n=4) | COL2A1; COL10A1; RUNX2; OCN; OPN; Proteoglycan / matrix; miR-1; miR-140; miR-26b | Integrates ISS and ECM-defect evidence with miRNA/exosome/signalling domains; mapped biology centers on miRNA regulation of proliferation, hypertrophy, ECM synthesis, and ossification. |
| (Liu et al, 2023) | ISS Themes: Exosome; Signalling; Growth plate | Outcome: Chondrocytes / cell culture; Exosomes; Growth plate tissue; Human / child samples; Rat model Inventory: Human; Animal; Chondrocyte culture; Omics | Outcome transitions: Proliferation; Hypertrophy; ECM maturation; Ossification Inventory readouts: Growth-plate phenotype; Proliferation; Hypertrophy; ECM maturation; Ossification | Decreased (n=8); Increased (n=1); Mixed / bidirectional (n=2); Descriptive / unclear (n=11) | RUNX2; OCN; OPN; ALP; Growth phenotype readout; Bone length | Integrates ISS outcome data with Exosome; Signalling; Growth plate evidence domains; mapped biology centers on Proliferation; Hypertrophy; ECM maturation; Ossification. |
| (Holling et al, 2023) | ECM defect; GH-related disorder; Related short-stature disorder Themes: Signalling; Growth plate | Outcome: Human / child samples; Unclear; Chondrocytes / cell culture; Growth plate tissue Inventory: Human; Omics; Animal; Chondrocyte culture | Outcome transitions: Proliferation; Hypertrophy; ECM maturation; Ossification; Growth-plate state transition not directly specified Inventory readouts: Growth-plate phenotype; Ossification; Proliferation | Increased (n=1); Descriptive / unclear (n=7) | Bone length; Proteoglycan / matrix; COL10A1; RUNX2; Growth phenotype readout | Integrates ECM defect; GH-related disorder; Related short-stature disorder outcome data with Signalling; Growth plate evidence domains; mapped biology centers on Proliferation; Hypertrophy; ECM maturation; Ossification; Growth-plate state transition not directly specified. |
| (Zieba et al, 2023) | ECM defect; GH-related disorder; Related growth-plate disorder Themes: Signalling; Growth plate | Outcome: Chondrocytes / cell culture; Growth plate tissue; Rat model Inventory: Animal; Chondrocyte culture; Omics; NR | Outcome transitions: Proliferation; Hypertrophy; ECM maturation; Ossification Inventory readouts: Growth-plate phenotype; Proliferation; ECM maturation; Hypertrophy; Ossification | Decreased (n=1); Increased (n=3); Mixed / bidirectional (n=2); Descriptive / unclear (n=7) | Proteoglycan / matrix; Bone length; COL2A1; COL10A1; RUNX2; Growth phenotype readout | Integrates ECM defect; GH-related disorder; Related growth-plate disorder outcome data with Signalling; Growth plate evidence domains; mapped biology centers on Proliferation; Hypertrophy; ECM maturation; Ossification. |
| (Mongiat et al, 2000) | ECM defect Themes: Signalling; Growth plate | Outcome: Chondrocytes / cell culture; Growth plate tissue; Unclear Inventory: Animal; NR; Chondrocyte culture | Outcome transitions: Hypertrophy; ECM maturation; Ossification; Proliferation Inventory readouts: Growth-plate phenotype; ECM maturation; Ossification; Hypertrophy | Increased (n=2); Descriptive / unclear (n=3) | Mineralization; Proteoglycan / matrix; RUNX2 | Integrates ECM defect outcome data with Signalling; Growth plate evidence domains; mapped biology centers on Hypertrophy; ECM maturation; Ossification; Proliferation. |
| (Starrett et al, 2025) | ECM defect; GH-related disorder Themes: Signalling; Growth plate | Outcome: Chondrocytes / cell culture; Growth plate tissue; Mouse model; Unclear Inventory: Animal; Chondrocyte culture | Outcome transitions: Proliferation; Hypertrophy; ECM maturation; Ossification; Growth-plate state transition not directly specified Inventory readouts: Growth-plate phenotype; Ossification; ECM maturation | Descriptive / unclear (n=9) | Bone length; Growth phenotype readout | Integrates ECM defect; GH-related disorder outcome data with Signalling; Growth plate evidence domains; mapped biology centers on Proliferation; Hypertrophy; ECM maturation; Ossification; Growth-plate state transition not directly specified. |
| (Nevarez et al, 2026) | ECM defect; GH-related disorder; Related short-stature disorder Themes: Signalling; Growth plate | Outcome: Chondrocytes / cell culture; Growth plate tissue; Mouse model Inventory: Human; Animal; Chondrocyte culture; Omics | Outcome transitions: Hypertrophy; ECM maturation; Ossification; Proliferation; Growth-plate state transition not directly specified Inventory readouts: Growth-plate phenotype; ECM maturation; Ossification; Hypertrophy | Decreased (n=3); Increased (n=1); Descriptive / unclear (n=8) | COL2A1; COL10A1; Mineralization; Bone length; Growth phenotype readout; Proteoglycan / matrix | Integrates ECM defect; GH-related disorder; Related short-stature disorder outcome data with Signalling; Growth plate evidence domains; mapped biology centers on Hypertrophy; ECM maturation; Ossification; Proliferation; Growth-plate state transition not directly specified. |
| (Bao et al, 2025) | ECM defect Themes: Signalling; Growth plate | Outcome: Human / child samples; Unclear Inventory: Omics; Human; NR | Outcome transitions: Proliferation; Hypertrophy; ECM maturation; Ossification Inventory readouts: Growth-plate phenotype | Descriptive / unclear (n=2) | COL2A1; COL10A1; Proteoglycan / matrix | Integrates ECM defect outcome data with Signalling; Growth plate evidence domains; mapped biology centers on Proliferation; Hypertrophy; ECM maturation; Ossification. |
| (Chu et al, 2026) | ECM defect; GH-related disorder Themes: Signalling; Growth plate | Outcome: Chondrocytes / cell culture; Growth plate tissue; Human / child samples; Rat model; Unclear Inventory: Human; Animal; Chondrocyte culture; Omics | Outcome transitions: Proliferation; Hypertrophy; ECM maturation; Ossification; Growth-plate state transition not directly specified Inventory readouts: Growth-plate phenotype; Proliferation; Ossification; Hypertrophy; ECM maturation | Decreased (n=1); Increased (n=3); Mixed / bidirectional (n=1); Descriptive / unclear (n=22) | Proteoglycan / matrix; Growth phenotype readout; COL2A1; COL10A1 | Integrates ECM defect; GH-related disorder outcome data with Signalling; Growth plate evidence domains; mapped biology centers on Proliferation; Hypertrophy; ECM maturation; Ossification; Growth-plate state transition not directly specified. |
| (Zhang et al, 2023) | ECM defect Themes: Growth plate | Outcome: Chondrocytes / cell culture; Growth plate tissue; Mouse model; Rat model Inventory: Human; Chondrocyte culture; Omics; Animal | Outcome transitions: Proliferation; ECM maturation; Ossification Inventory readouts: Ossification; Proliferation; ECM maturation; Growth-plate phenotype | Decreased (n=1); Increased (n=2); Mixed / bidirectional (n=1); Descriptive / unclear (n=5) | Bone length; Body length; Proteoglycan / matrix; RUNX2; Mineralization | Integrates ECM defect outcome data with Growth plate evidence domains; mapped biology centers on Proliferation; ECM maturation; Ossification. |
| (Trompet et al, 2024) | ECM defect; GH-related disorder; Related growth-plate disorder Themes: Signalling; Growth plate | Outcome: Growth plate tissue; Rat model; Chondrocytes / cell culture; Mouse model; Unclear Inventory: Animal; Chondrocyte culture; Omics | Outcome transitions: Proliferation; Hypertrophy; Ossification Inventory readouts: Growth-plate phenotype; Ossification; Proliferation | Decreased (n=2); Increased (n=2); Mixed / bidirectional (n=1); Descriptive / unclear (n=16) | Bone length; Growth phenotype readout | Integrates ECM defect; GH-related disorder; Related growth-plate disorder outcome data with Signalling; Growth plate evidence domains; mapped biology centers on Proliferation; Hypertrophy; Ossification. |
| (Pan et al, 2026) | ECM defect; GH-related disorder Themes: Exosome; Growth plate | Outcome: Chondrocytes / cell culture; Exosomes; Growth plate tissue; Mouse model; Unclear Inventory: Animal; Chondrocyte culture; Engineered exosome; NR; Omics | Outcome transitions: Proliferation; ECM maturation; Ossification; Hypertrophy Inventory readouts: Growth-plate phenotype; ECM maturation; Ossification; Hypertrophy | Decreased (n=2); Increased (n=1); Descriptive / unclear (n=11) | COL2A1; Proteoglycan / matrix; COL10A1; Growth-plate thickness; Body length | Integrates ECM defect; GH-related disorder outcome data with Exosome; Growth plate evidence domains; mapped biology centers on Proliferation; ECM maturation; Ossification; Hypertrophy. |
| (Yu et al, 2024) | ECM defect; GH-related disorder Themes: Signalling; Growth plate | Outcome: Growth plate tissue; Mouse model; Chondrocytes / cell culture; Rat model; Unclear Inventory: Animal; Omics; Chondrocyte culture | Outcome transitions: Proliferation; Hypertrophy; ECM maturation; Ossification Inventory readouts: Ossification; ECM maturation; Hypertrophy; Proliferation | Decreased (n=1); Mixed / bidirectional (n=1); Descriptive / unclear (n=8) | OCN; Proteoglycan / matrix; COL2A1; Mineralization; Body length | Integrates ECM defect; GH-related disorder outcome data with Signalling; Growth plate evidence domains; mapped biology centers on Proliferation; Hypertrophy; ECM maturation; Ossification. |
| (Tsukamoto et al, 2023) | ECM defect; GH-related disorder; Related short-stature disorder Themes: Signalling; Growth plate | Outcome: Chondrocytes / cell culture; Growth plate tissue; Unclear; Mouse model Inventory: Animal; Chondrocyte culture | Outcome transitions: Hypertrophy; Ossification; ECM maturation Inventory readouts: Ossification; Hypertrophy | Decreased (n=1); Increased (n=2); Descriptive / unclear (n=9) | OCN; Mineralization; Bone length | Integrates ECM defect; GH-related disorder; Related short-stature disorder outcome data with Signalling; Growth plate evidence domains; mapped biology centers on Hypertrophy; Ossification; ECM maturation. |
| (Zhou et al, 2024) | ECM defect; GH-related disorder; Related growth-plate disorder Themes: Signalling; Growth plate | Outcome: Growth plate tissue; Human / child samples; Mouse model; Unclear; Chondrocytes / cell culture Inventory: Animal; Chondrocyte culture; Human | Outcome transitions: Proliferation; Hypertrophy; ECM maturation; Ossification Inventory readouts: Ossification; Hypertrophy; ECM maturation | Descriptive / unclear (n=12) | Growth phenotype readout; COL2A1; COL10A1; Mineralization; RUNX2 | Integrates ECM defect; GH-related disorder; Related growth-plate disorder outcome data with Signalling; Growth plate evidence domains; mapped biology centers on Proliferation; Hypertrophy; ECM maturation; Ossification. |
| (Rashid et al, 2024) | ECM defect Themes: Signalling; Growth plate | Outcome: Chondrocytes / cell culture; Growth plate tissue; Mouse model; Unclear Inventory: Animal; Chondrocyte culture | Outcome transitions: Hypertrophy; ECM maturation; Ossification Inventory readouts: Ossification; Hypertrophy; ECM maturation | Descriptive / unclear (n=4) | COL10A1; RUNX2; COL2A1; Mineralization; OCN; OPN | Integrates ECM defect outcome data with Signalling; Growth plate evidence domains; mapped biology centers on Hypertrophy; ECM maturation; Ossification. |
| (Zhang et al, 2024) | ECM defect; GH-related disorder Themes: Signalling; Growth plate | Outcome: Chondrocytes / cell culture; Growth plate tissue; Mouse model; Unclear Inventory: Animal; Chondrocyte culture | Outcome transitions: Proliferation; Hypertrophy; ECM maturation; Ossification Inventory readouts: Proliferation; Ossification | Decreased (n=1); Descriptive / unclear (n=6) | RUNX2; Bone length; Body length; Growth phenotype readout | Integrates ECM defect; GH-related disorder outcome data with Signalling; Growth plate evidence domains; mapped biology centers on Proliferation; Hypertrophy; ECM maturation; Ossification. |
| (Taieb et al, 2023) | ECM defect Themes: ECM; Signalling; Growth plate | Outcome: Chondrocytes / cell culture; Growth plate tissue; Human / child samples; Mouse model Inventory: Human; Animal; Chondrocyte culture | Outcome transitions: Hypertrophy; ECM maturation; Ossification Inventory readouts: Hypertrophy; ECM maturation; Ossification | Decreased (n=1); Increased (n=1); Descriptive / unclear (n=5) | OCN; Mineralization; Body length; COL2A1; COL10A1; RUNX2; OPN; XylT-I; proteoglycans; glycosaminoglycan chains | Integrates ECM-defect outcome data with ECM/signalling/growth-plate domains; mapped biology centers on proteoglycan synthesis, collagen organization, chondrocyte maturation, and matrix-dependent ossification. |
| (Yuan et al, 2023) | ECM defect; GH-related disorder; Related short-stature disorder Themes: Growth plate | Outcome: Chondrocytes / cell culture; Growth plate tissue Inventory: Animal; Chondrocyte culture | Outcome transitions: Proliferation; Hypertrophy; ECM maturation; Ossification Inventory readouts: Proliferation; Ossification | Increased (n=1); Descriptive / unclear (n=5) | Proteoglycan / matrix; Growth phenotype readout; COL10A1; Bone length; Body length | Integrates ECM defect; GH-related disorder; Related short-stature disorder outcome data with Growth plate evidence domains; mapped biology centers on Proliferation; Hypertrophy; ECM maturation; Ossification. |
| (Baronas et al, 2023) | GH-related disorder; Related growth-plate disorder Themes: Signalling; Growth plate | Outcome: Chondrocytes / cell culture; Rat model; Unclear Inventory: Animal; Chondrocyte culture; Omics | Outcome transitions: Proliferation; Hypertrophy; Ossification Inventory readouts: Proliferation; Growth-plate phenotype | Increased (n=3); Mixed / bidirectional (n=1); Descriptive / unclear (n=7) | Growth phenotype readout; COL2A1; RUNX2; Phenotype endpoint not directly specified | Integrates GH-related disorder; Related growth-plate disorder outcome data with Signalling; Growth plate evidence domains; mapped biology centers on Proliferation; Hypertrophy; Ossification. |
| (Yokoyama et al, 2024) | GH-related disorder Themes: Growth plate | Outcome: Chondrocytes / cell culture; Growth plate tissue Inventory: Animal; Chondrocyte culture | Outcome transitions: Proliferation; Hypertrophy; Ossification Inventory readouts: Hypertrophy; Ossification; Proliferation | Descriptive / unclear (n=2) | Growth phenotype readout | Integrates GH-related disorder outcome data with Growth plate evidence domains; mapped biology centers on Proliferation; Hypertrophy; Ossification. |
| (Weaver et al, 2024) | Related growth-plate disorder Themes: Growth plate | Outcome: Chondrocytes / cell culture; Growth plate tissue; Mouse model; Rat model; Unclear Inventory: Animal; Chondrocyte culture; Omics | Outcome transitions: Hypertrophy; Ossification Inventory readouts: Ossification | Mixed / bidirectional (n=1); Descriptive / unclear (n=5) | Mineralization; Body length | Integrates Related growth-plate disorder outcome data with Growth plate evidence domains; mapped biology centers on Hypertrophy; Ossification. |
| (Cho et al, 2024) | Related growth-plate disorder; short stature; growth-plate closure Themes: Growth plate; therapeutic intervention | Outcome: Human growth-plate biology; therapeutic intervention literature; review-based synthesis Inventory: Human; Review; Therapeutic intervention | Outcome transitions: Proliferation; Hypertrophy; Ossification; Growth-plate senescence; Growth-plate closure Inventory readouts: Growth-plate phenotype; Ossification; Growth-plate closure | Descriptive / unclear | Growth-plate zones; chondrocyte proliferation; hypertrophic chondrocytes; calcification; vascularization; growth-plate closure | Integrates growth-plate closure and therapeutic-intervention evidence; mapped biology centers on proliferation, hypertrophy, ossification, senescence, and closure of the growth plate. |
| (Liu et al, 2021) | ISS Themes: Signalling; Growth plate | Outcome: Chondrocytes / cell culture; Growth plate tissue; Mouse model Inventory: Human; Animal; Chondrocyte culture; Omics | Outcome transitions: Proliferation; Hypertrophy; Ossification; ECM maturation Inventory readouts: Proliferation; Hypertrophy; Ossification; Mineralization | Decreased / dysregulated | COL10A1; RUNX2; OCN; OPN; ALP; Mineralization; circANAPC2; miR-874-3p; SMAD3 | Integrates ISS signalling and growth-plate evidence; mapped biology centers on circANAPC2-mediated suppression of chondrocyte growth, hypertrophy, mineralization, and endochondral ossification. |
| (Whitaker et al, 2017) | ECM defect; Related short-stature disorder Themes: Signalling; Growth plate; ECM | Outcome: Chondrocytes / cell culture; Growth plate tissue; Mouse model Inventory: Animal; Chondrocyte culture | Outcome transitions: Proliferation; Hypertrophy; ECM maturation Inventory readouts: ECM maturation; Proliferation; Hypertrophy; Growth-plate phenotype | Decreased / dysregulated | Smad4; aggrecan; MMP13; chondrocyte polarity; proliferation; hypertrophy; ECM production | Integrates ECM and growth-plate evidence; mapped biology centers on Smad4-dependent regulation of matrix production, chondrocyte polarity, proliferation, and hypertrophy. |
| (Kang, 2017) | ISS Themes: Genetic cause; GH–IGF axis; Growth plate | Outcome: Human / child genetic evidence; review-based synthesis Inventory: Human; Review; Genetic diagnosis | Outcome transitions: Growth-plate state transition not directly specified; ECM maturation; Paracrine signalling Inventory readouts: Growth-plate phenotype; Genetic endpoint | Descriptive / unclear | GHR; IGFALS; SHOX; NPR2; NPPC; FGFR3; ACAN | Integrates ISS genetic evidence with GH–IGF and growth-plate domains; mapped biology centers on monogenic causes involving endocrine, paracrine, ECM, and chondrocyte-intrinsic mechanisms. |
| (Baron et al, 2015) | Short stature; Related growth-plate disorder Themes: Growth plate; endocrine/paracrine signalling; ECM | Outcome: Human genetic evidence; growth-plate biology; review-based synthesis Inventory: Human; Review; Genetic evidence | Outcome transitions: Proliferation; Hypertrophy; ECM maturation; Ossification Inventory readouts: Growth-plate phenotype; Chondrogenesis; ECM regulation | Descriptive / unclear | GH; IGF-1; growth-plate chondrocytes; ECM molecules; paracrine factors; intracellular proteins | Integrates short- and tall-stature evidence into a growth-plate-centered framework; mapped biology centers on chondrocyte regulation by endocrine, paracrine, ECM, and intracellular mechanisms. |
| (Kim, 2022) | ISS; Related short-stature disorder Themes: Genetic diagnosis; Growth plate; GH–IGF axis | Outcome: Human / child samples; genetic diagnosis literature; review-based synthesis Inventory: Human; Review; Genetic testing | Outcome transitions: Growth-plate state transition not directly specified; ECM maturation; Paracrine signalling Inventory readouts: Growth-plate phenotype; Genetic endpoint | Descriptive / unclear | Genetic testing; skeletal survey; endocrine evaluation; pituitary–IGF axis; growth-plate genes | Integrates pediatric short-stature genetic evidence with growth-plate and endocrine domains; mapped biology centers on growth-plate disruption and pituitary–IGF axis defects. |
| (Bjarnason et al, 2004) | GH-related disorder; Short stature Themes: GH response; ECM; Growth plate | Outcome: Chondrocytes / cell culture; Human / child samples; GH-treated short children Inventory: Human; Chondrocyte culture; Clinical cohort | Outcome transitions: ECM maturation; Growth response; Growth-plate state transition not directly specified Inventory readouts: ECM maturation; Growth phenotype | Increased after GH treatment | COMP; GH; IGF-I; serum COMP; height SDS; short prepubertal children | Integrates GH-related short-stature evidence with ECM response domains; mapped biology centers on GH/IGF-I-mediated induction of cartilage oligomeric matrix protein and serum ECM-marker response during GH treatment. |
| (Kodama et al, 2020) | ECM defect; Related short-stature disorder Themes: ECM; Growth plate | Outcome: Growth plate tissue; Mouse model Inventory: Animal; Growth-plate tissue | Outcome transitions: ECM maturation; Hypertrophy; Chondrocyte differentiation; Growth-plate phenotype Inventory readouts: ECM maturation; Hypertrophy; Growth phenotype | Decreased / impaired | CANT1; glycosaminoglycans; ECM space; proliferating chondrocytes; hypertrophic chondrocytes; dwarf phenotype | Integrates Desbuquois dysplasia model evidence with ECM/growth-plate domains; mapped biology centers on impaired GAG synthesis, reduced ECM space, and defective hypertrophic differentiation. |
| (Kim et al, 2023) | ISS; Familial short stature; GH-related disorder Themes: Genetic diagnosis; GH response; Growth plate | Outcome: Human / child samples; targeted exome sequencing; GH-treated cases Inventory: Human; Omics; Clinical cohort | Outcome transitions: Growth-plate state transition not directly specified; ECM maturation; Paracrine signalling; GH response Inventory readouts: Growth phenotype; Genetic endpoint; Height SDS | Descriptive / unclear; improved height after GH in subset | NPR2; IHH; COL1A1; COL2A1; TRPS1; SPRED1; PTPN11; diagnostic yield 41.7%; height SDS | Integrates ISS/FSS genetic and GH-response evidence; mapped biology centers on growth-plate, ECM, paracrine, and intracellular genes associated with postnatal growth failure. |
| (Ono et al, 2013) | Related growth-plate disorder Themes: FGFR/RAS–ERK signalling; Growth plate | Outcome: Chondrocytes / cell culture; Growth plate tissue; Mouse model Inventory: Animal; Chondrocyte culture | Outcome transitions: Proliferation; Hypertrophy; Ossification; Chondrocyte maturation Inventory readouts: Proliferation; Growth-plate phenotype; Ossification | Decreased proliferation/maturation; increased ERK activation | NF1; ERK1/2; FGFR; IHH; RANKL; MMP9; MMP13; CNP; short stature | Integrates related growth-plate disorder evidence with signalling domains; mapped biology centers on neurofibromin-mediated restraint of ERK-dependent FGFR signalling during endochondral bone formation. |
| (Taye et al, 2025) | ECM defect; Related short-stature disorder Themes: ECM; Growth plate | Outcome: Growth plate tissue; Mouse model; fibroblast ECM assays Inventory: Animal; Chondrocyte-related tissue | Outcome transitions: Hypertrophy; ECM maturation; Ossification; Growth-plate phenotype Inventory readouts: Bone length; Hypertrophic zone; ECM secretion/assembly | Decreased / dysregulated | ADAMTS10; ADAMTS17; fibronectin; fibrillin-1; hypertrophic zone; bone shortening | Integrates final ADAMTS10/ADAMTS17 publication evidence with ECM/growth-plate domains; mapped biology centers on ECM secretion or pericellular matrix assembly and bone shortening. |
| (Ağırdil, 2020) | Related growth-plate disorder; growth-plate physiology Themes: Growth plate | Outcome: Growth-plate biology; review-based synthesis Inventory: Review; Human physiology | Outcome transitions: Proliferation; Hypertrophy; Ossification; Growth-plate closure Inventory readouts: Growth-plate phenotype; Ossification | Descriptive / unclear | Resting zone; proliferative zone; hypertrophic zone; collagen matrix; osteoblast replacement; longitudinal growth | Integrates physiological growth-plate evidence; mapped biology centers on coordinated chondrocyte proliferation, hypertrophy, matrix replacement, ossification, and growth-plate closure. |
| (Saltarelli et al, 2022) | ECM defect; Related short-stature disorder Themes: ECM; Growth plate; genetic diagnosis | Outcome: Human / child samples; genetic ECM disorders; review-based synthesis Inventory: Human; Review; Genetic evidence | Outcome transitions: ECM maturation; Chondrogenesis; Growth-plate state transition not directly specified Inventory readouts: ECM maturation; Growth-plate phenotype; Genetic endpoint | Descriptive / unclear | ECM genes; growth-plate organization; intracellular signalling; paracrine signalling; endocrine signalling | Integrates ECM-defect and short-stature evidence; mapped biology centers on genetic defects affecting ECM organization, chondrogenesis, and growth-plate function. |

**Table S2.** **Molecular route–phenotype evidence synthesis**

| **Molecular / pathway cluster** | **Representative molecules or readouts** | **Source evidence** | **Phenotype and direction profile** | **Evidence support** | **Main gap(s)** | **Integrated interpretation** |
| --- | --- | --- | --- | --- | --- | --- |
| miR-26b-3p–AKAP2 exosomal cargo axis | miR-26b-3p; AKAP2; ALP; OCN; OPN; RUNX2; BrdU; MiR-26B-3P; +5 more | 2 source-study label(s): (Liu et al, 2023); (Thakore & Delany, 2025) | Phenotypes: Proliferation (19); Hypertrophy (4); ECM maturation (3); Ossification (17); Growth-plate phenotype (3) Direction: Decreased (15); Increased (2); Rescued (4); Altered / unclear (4) | Human validation (12); In vivo (22); Functional assay (16); Rescue (4); Association (1) | Causal rescue of exosomal cargo effect remains limited. (15); Direct cargo-to-target validation remains untested. (9); +1 more | Exosomal cargo/readout evidence links this cluster mainly to proliferation, ossification, hypertrophy; the pattern is decreased, but cargo-to-target and rescue evidence remain uneven. |
| miR-17-3p / ERK–RUNX2 exosomal axis | miR-17-3p; miR-17; RUNX2; OCN; OPN; miR-17-3p-oeGH; ALP; miR-17-3p-induced; +5 more | 1 source-study label(s): (Wang et al, 2025) | Phenotypes: Proliferation (21); Hypertrophy (3); Ossification (8); Growth-plate phenotype (2) Direction: Decreased (5); Increased (2); Rescued (2); Altered / unclear (14) | Human validation (6); In vivo (22); Functional assay (11); Rescue (3); Association (1) | Direct cargo-to-target validation remains untested. (12); Causal rescue of exosomal cargo effect remains limited. (10); +1 more | Exosomal cargo/readout evidence links this cluster mainly to proliferation, ossification, hypertrophy; the pattern is altered / unclear, but cargo-to-target and rescue evidence remain uneven. |
| miR-185-3p–MAPK1 exosomal axis | miR-185-3p; MAPK1; ALP; OCN; OPN; RUNX2; Ocn; Opn; +2 more | 1 source-study label(s): (Wu et al, 2022) | Phenotypes: Proliferation (16); Hypertrophy (9); ECM maturation (4); Ossification (17); Growth-plate phenotype (2) Direction: Decreased (5); Increased (2); Rescued (1); Altered / unclear (13) | Human validation (10); In vivo (17); Functional assay (9); Rescue (1); Association (3) | Direct cargo-to-target validation remains untested. (12); Causal rescue of exosomal cargo effect remains limited. (8); +1 more | Exosomal cargo/readout evidence links this cluster mainly to ossification, proliferation, hypertrophy; the pattern is altered / unclear, but cargo-to-target and rescue evidence remain uneven. |
| Unspecified exosomal cargo linked to proliferation–ossification | Not clearly stated / phenotype-level | 4 source-study label(s): (Liu et al, 2023); (Wang et al, 2025); (Yuan et al, 2024); (Wu et al, 2022) | Phenotypes: Proliferation (13); Hypertrophy (4); Ossification (13) Direction: Decreased (3); Increased (2); Rescued (3); Altered / unclear (5) | Human validation (5); In vivo (13); Functional assay (8); Rescue (4) | Causal rescue of exosomal cargo effect remains limited. (7); Direct cargo-to-target validation remains untested. (5); +1 more | Exosomal cargo/readout evidence links this cluster mainly to proliferation, ossification, hypertrophy; the pattern is altered / unclear, but cargo-to-target and rescue evidence remain uneven. |
| Unspecified exosomal cargo linked to ossification | Not clearly stated / phenotype-level | 5 source-study label(s): (Yuan et al, 2024); (Zieba et al, 2023); (Wang et al, 2025); (Liu et al, 2023); (Wu et al, 2022) | Phenotypes: Hypertrophy (1); Ossification (10) Direction: Decreased (2); Increased (3); Rescued (2); Altered / unclear (3) | Human validation (5); In vivo (4); Functional assay (1); Rescue (2); Association (3) | Direct cargo-to-target validation remains untested. (9); ECM-dependent exosome uptake remains untested. (1) | Exosomal cargo/readout evidence links this cluster mainly to ossification, hypertrophy; the pattern is increased, but cargo-to-target and rescue evidence remain uneven. |
| Exosomal circRNA/circular RNA signal | circular; circle; circRNA; Circos; Trpv4; Col2a1; Sox9; RUNX2; +4 more | 7 source-study label(s): (Nevarez et al, 2026); (Liu et al, 2020); (Zieba et al, 2023); (Pan et al, 2026); (Zhang et al, 2023); +2 more | Phenotypes: Proliferation (2); Hypertrophy (1); ECM maturation (2); Ossification (6); Growth-plate phenotype (2) Direction: Decreased (2); Increased (3); Rescued (1); Altered / unclear (3) | Human validation (2); In vivo (7); Functional assay (1); Rescue (1); Association (2) | Direct cargo-to-target validation remains untested. (8); Causal rescue of exosomal cargo effect remains limited. (1) | Exosomal cargo/readout evidence links this cluster mainly to ossification, ecm maturation, proliferation; the pattern is increased, but cargo-to-target and rescue evidence remain uneven. |
| Osteogenic/proliferative readout panel within exosomal studies | ALP; OCN; OPN; RUNX2; Runx2; Ocn; Opn | 3 source-study label(s): (Yuan et al, 2024); (Wu et al, 2022); (Wang et al, 2025) | Phenotypes: Proliferation (8); Hypertrophy (7); ECM maturation (2); Ossification (7) Direction: Decreased (6); Increased (1); Altered / unclear (2) | Human validation (4); In vivo (9); Functional assay (5) | Causal rescue of exosomal cargo effect remains limited. (5); Direct cargo-to-target validation remains untested. (4) | Exosomal cargo/readout evidence links this cluster mainly to proliferation, ossification, hypertrophy; the pattern is decreased, but cargo-to-target and rescue evidence remain uneven. |
| Unspecified exosomal cargo linked to proliferation | Not clearly stated / phenotype-level | 2 source-study label(s): (Yuan et al, 2024); (Wang et al, 2025) | Phenotypes: Proliferation (9) Direction: Decreased (2); Increased (3); Rescued (1); Altered / unclear (3) | Human validation (8); In vivo (9); Functional assay (3); Rescue (2) | Direct cargo-to-target validation remains untested. (6); Causal rescue of exosomal cargo effect remains limited. (3) | Exosomal cargo/readout evidence links this cluster mainly to proliferation; the pattern is increased, but cargo-to-target and rescue evidence remain uneven. |
| Unspecified exosomal cargo linked to growth-plate phenotype | Not clearly stated / phenotype-level | 6 source-study label(s): (Wu et al, 2022); (Liu et al, 2023); (Wang et al, 2025); (Thakore & Delany, 2025); (Shin et al, 2026); +1 more | Phenotypes: Growth-plate phenotype (7) Direction: Impaired (1); Altered / unclear (6) | Human validation (2); In vivo (3); Functional assay (3); Association (1) | Direct cargo-to-target validation remains untested. (4); Causal rescue of exosomal cargo effect remains limited. (3) | Exosomal cargo/readout evidence links this cluster mainly to growth-plate phenotype; the pattern is altered / unclear, but cargo-to-target and rescue evidence remain uneven. |
| miR-877-3p / miR-4691-5p / miR-939-3p exosomal axis | miR-877-3p; miR-4691-5p; miR-939-3p | 1 source-study label(s): (Yuan et al, 2024) | Phenotypes: Proliferation (4); Ossification (2) Direction: Decreased (3); Increased (2); Rescued (1) | Human validation (6); In vivo (4); Functional assay (3); Rescue (2) | Causal rescue of exosomal cargo effect remains limited. (3); Direct cargo-to-target validation remains untested. (3) | Exosomal cargo/readout evidence links this cluster mainly to proliferation, ossification; the pattern is decreased, but cargo-to-target and rescue evidence remain uneven. |
| Exosome-marker / vesicle-level readouts | CD9; CD63 | 4 source-study label(s): (Yuan et al, 2024); (Wang et al, 2025); (Liu et al, 2023); (Pan et al, 2026) | Phenotypes: Proliferation (2); Growth-plate phenotype (2) Direction: Decreased (1); Altered / unclear (3) | Human validation (3); In vivo (3); Functional assay (1) | Direct cargo-to-target validation remains untested. (3); Causal rescue of exosomal cargo effect remains limited. (1) | Exosomal cargo/readout evidence links this cluster mainly to proliferation, growth-plate phenotype; the pattern is altered / unclear, but cargo-to-target and rescue evidence remain uneven. |
| Other exosomal miRNA signatures | miR-4488 | 1 source-study label(s): (Shin et al, 2026) | Phenotypes: Growth-plate phenotype (1) Direction: Decreased (1) | Human validation (1); In vivo (1) | Direct cargo-to-target validation remains untested. (1) | Exosomal cargo/readout evidence links this cluster mainly to growth-plate phenotype; the pattern is decreased, but cargo-to-target and rescue evidence remain uneven. |
| **Route category: ECM defect — matrix structure, hypertrophy, and endochondral progression \| 4 consolidated cluster row(s); 34 extracted row(s)** | | | | | | |
| Collagen–SOX9 matrix architecture module | Sox9; Col2a1; Col10; DAPI | 3 source-study label(s): (Taieb et al, 2023); (Nevarez et al, 2026); (Zieba et al, 2023) | Phenotypes: Hypertrophy (8); ECM maturation (13); Ossification (10) Direction: Increased (6); Impaired (1); Altered / unclear (6) | In vivo (10); Functional assay (1); Association (3) | Matrix-rescue experiments remain limited. (10); In vivo linkage between matrix defect and phenotype remains limited. (3) | Matrix-associated changes concentrate on ecm maturation, ossification, hypertrophy, supporting ECM architecture as a structural regulator of endochondral progression. |
| Matrix-level phenotype without named mediator | Not clearly stated / phenotype-level | 8 source-study label(s): (Taieb et al, 2023); (Zieba et al, 2023); (Starrett et al, 2025); (Nevarez et al, 2026); (Zhang et al, 2023); +3 more | Phenotypes: Proliferation (1); Hypertrophy (6); ECM maturation (13); Ossification (10) Direction: Decreased (2); Increased (4); Impaired (2); Altered / unclear (5) | Human validation (2); In vivo (10); Association (2) | Matrix-rescue experiments remain limited. (10); In vivo linkage between matrix defect and phenotype remains limited. (3) | Matrix-associated changes concentrate on ecm maturation, ossification, hypertrophy, supporting ECM architecture as a structural regulator of endochondral progression. |
| IHH–RUNX2 hypertrophy/ossification coupling | Ihh; Runx2; RUNX2; Col2a1; OCN; OPN; Sox9; ANP | 5 source-study label(s): (Taieb et al, 2023); (Rashid et al, 2024); (Yuan et al, 2023); (Liu et al, 2023); (Zhou et al, 2024) | Phenotypes: Proliferation (1); Hypertrophy (5); ECM maturation (5); Ossification (5) Direction: Decreased (1); Increased (2); Rescued (1); Altered / unclear (2) | Human validation (2); In vivo (3); Functional assay (1); Association (1) | Matrix-rescue experiments remain limited. (3); In vivo linkage between matrix defect and phenotype remains limited. (3) | Matrix-associated changes concentrate on hypertrophy, ecm maturation, ossification, supporting ECM architecture as a structural regulator of endochondral progression. |
| FGF19/SOX9-associated matrix signalling | FGF19; SOX9 | 1 source-study label(s): (Chen et al, 2025) | Phenotypes: ECM maturation (2); Ossification (2) Direction: Altered / unclear (2) | Association (2) | In vivo linkage between matrix defect and phenotype remains limited. (2) | Matrix-associated changes concentrate on ecm maturation, ossification, supporting ECM architecture as a structural regulator of endochondral progression. |
| **Route category: GH–IGF — systemic growth-axis phenotype and responsiveness \| 1 consolidated cluster row(s); 3 extracted row(s)** | | | | | | |
| GH–IGF responsiveness / growth-plate phenotype | Not clearly stated / phenotype-level | 2 source-study label(s): (Shin et al, 2026); (Chen et al, 2024) | Phenotypes: Growth-plate phenotype (3) Direction: Impaired (2); Altered / unclear (1) | Human validation (2); In vivo (3) | Mechanism-specific rescue stratification remains limited. (2); Prediction of GH responsiveness remains untested. (1) | GH–IGF rows emphasize impaired or unclear growth-plate phenotype and the need to stratify mechanism-specific treatment responsiveness. |
| **Route category: Local signalling — local pathway nodes and chondrocyte state transitions \| 16 consolidated cluster row(s); 138 extracted row(s)** | | | | | | |
| Unspecified local signalling linked to growth-plate phenotype | Not clearly stated / phenotype-level | 14 source-study label(s): (Holling et al, 2023); (Wu et al, 2022); (Nevarez et al, 2026); (Trompet et al, 2024); (Yuan et al, 2023); +9 more | Phenotypes: Growth-plate phenotype (21) Direction: Decreased (5); Increased (7); Impaired (1); Rescued (2); Altered / unclear (6) | Human validation (8); In vivo (10); Functional assay (7); Rescue (2); Association (3) | Pathway-causal testing remains limited. (14); Node-level rescue experiments remain limited. (7) | Local signalling nodes converge on growth-plate phenotype; evidence is strongest in vivo but pathway-causal and node-level rescue tests are still limited. |
| Proliferation/cell-state marker readouts | DAPI; BrdU; Ki67; Rheb1; PCNA; SOX9 | 6 source-study label(s): (Zhang et al, 2024); (Yuan et al, 2023); (Xiong et al, 2025); (Holling et al, 2023); (Trompet et al, 2024); +1 more | Phenotypes: Proliferation (12); Ossification (14); Growth-plate phenotype (2) Direction: Decreased (4); Increased (1); Impaired (3); Rescued (1); Altered / unclear (7) | Human validation (2); In vivo (15); Rescue (1) | Pathway-causal testing remains limited. (16) | Local signalling nodes converge on ossification, proliferation, growth-plate phenotype; evidence is strongest in vivo but pathway-causal and node-level rescue tests are still limited. |
| TGF-β / SMAD / BMP signalling | TGF-β; BMP; SOX9; SMAD2; circRNA; circular; RUNX2; IHH; +5 more | 5 source-study label(s): (Costantini et al, 2023); (Liu et al, 2020); (Chen et al, 2025); (Thakore & Delany, 2025); (Baronas et al, 2023) | Phenotypes: Proliferation (4); Hypertrophy (1); Ossification (5); Growth-plate phenotype (5) Direction: Decreased (3); Increased (3); Altered / unclear (7) | In vivo (13); Functional assay (1) | Pathway-causal testing remains limited. (12); Node-level rescue experiments remain limited. (1) | Local signalling nodes converge on growth-plate phenotype, ossification, proliferation; evidence is strongest in vivo but pathway-causal and node-level rescue tests are still limited. |
| FGF/FGFR local signalling | FGF19; SOX9; FGFR3; Fgfr3; ALP; PTHrP; Ihh; IGF-1 | 4 source-study label(s): (Chen et al, 2025); (Starrett et al, 2025); (Taieb et al, 2023); (Saltarelli et al, 2022) | Phenotypes: Proliferation (3); Hypertrophy (3); ECM maturation (1); Ossification (9); Growth-plate phenotype (1) Direction: Decreased (3); Increased (2); Rescued (1); Altered / unclear (6) | In vivo (10); Rescue (2); Association (1) | Pathway-causal testing remains limited. (12) | Local signalling nodes converge on ossification, proliferation, hypertrophy; evidence is strongest in vivo but pathway-causal and node-level rescue tests are still limited. |
| RUNX2/SOX9/osteochondral readout module | SOX9; OCN; COL2A1; OPN; Runx2; Sox9; Col2a1 | 7 source-study label(s): (Xiong et al, 2025); (Saltarelli et al, 2022); (Yu et al, 2024); (Liu et al, 2023); (Rashid et al, 2024); +2 more | Phenotypes: Proliferation (3); Hypertrophy (3); ECM maturation (2); Ossification (8) Direction: Decreased (1); Increased (3); Altered / unclear (6) | Human validation (3); In vivo (8); Association (1) | Pathway-causal testing remains limited. (10) | Local signalling nodes converge on ossification, proliferation, hypertrophy; evidence is strongest in vivo but pathway-causal and node-level rescue tests are still limited. |
| ANP–corin hypertrophy/ossification axis | ANP; corin; Corin; Col10; Col2a1 | 1 source-study label(s): (Zhou et al, 2024) | Phenotypes: Hypertrophy (6); ECM maturation (2); Ossification (9) Direction: Altered / unclear (9) | In vivo (8); Functional assay (1) | Pathway-causal testing remains limited. (8); Node-level rescue experiments remain limited. (1) | Local signalling nodes converge on ossification, hypertrophy, ecm maturation; evidence is strongest in vivo but pathway-causal and node-level rescue tests are still limited. |
| Unspecified local signalling linked to ossification | Not clearly stated / phenotype-level | 9 source-study label(s): (Xiong et al, 2025); (Starrett et al, 2025); (Holling et al, 2023); (Zhou et al, 2024); (Zieba et al, 2023); +4 more | Phenotypes: ECM maturation (1); Ossification (9) Direction: Decreased (1); Increased (2); Rescued (1); Altered / unclear (5) | Human validation (3); In vivo (6); Functional assay (2); Association (1) | Pathway-causal testing remains limited. (7); Node-level rescue experiments remain limited. (2) | Local signalling nodes converge on ossification, ecm maturation; evidence is strongest in vivo but pathway-causal and node-level rescue tests are still limited. |
| miRNA-linked local signalling | miR-133a-3p; miR-335-5p; miR-26b-3p; SOX9; miR-322-5p; miR-140; miR-143-3p; Sox9; +5 more | 3 source-study label(s): (Thakore & Delany, 2025); (Shin et al, 2026); (Liu et al, 2023) | Phenotypes: Hypertrophy (1); Growth-plate phenotype (7) Direction: Decreased (2); Increased (4); Altered / unclear (2) | Human validation (4); In vivo (3); Functional assay (2); Association (1) | Pathway-causal testing remains limited. (6); Node-level rescue experiments remain limited. (2) | Local signalling nodes converge on growth-plate phenotype, hypertrophy; evidence is strongest in vivo but pathway-causal and node-level rescue tests are still limited. |
| MAPK/ERK signalling node | ERK1/2; MAPK1; miR-26b-3p; miR-185-3p; miR-26b; Akap2 | 4 source-study label(s): (Wu et al, 2022); (Liu et al, 2023); (Taieb et al, 2023); (Thakore & Delany, 2025) | Phenotypes: Hypertrophy (2); ECM maturation (1); Growth-plate phenotype (5) Direction: Decreased (2); Rescued (1); Altered / unclear (4) | Human validation (4); In vivo (1); Functional assay (4); Rescue (1); Association (1) | Pathway-causal testing remains limited. (3); Node-level rescue experiments remain limited. (3); +1 more | Local signalling nodes converge on growth-plate phenotype, hypertrophy, ecm maturation; evidence is strongest in vivo but pathway-causal and node-level rescue tests are still limited. |
| Unspecified local signalling linked to proliferation–ossification | Not clearly stated / phenotype-level | 7 source-study label(s): (Zhang et al, 2023); (Xiong et al, 2025); (Trompet et al, 2024); (Yuan et al, 2023); (Holling et al, 2023); +2 more | Phenotypes: Proliferation (7); Hypertrophy (1); Ossification (7) Direction: Decreased (1); Increased (2); Impaired (1); Altered / unclear (3) | Human validation (2); In vivo (7) | Pathway-causal testing remains limited. (7) | Local signalling nodes converge on proliferation, ossification, hypertrophy; evidence is strongest in vivo but pathway-causal and node-level rescue tests are still limited. |
| IHH–PTHrP regulatory axis | Ihh; Pthrp; IHH; PTHrP; miR-503-5p; miR-374-5p; miR-379-5p; miR-1; +2 more | 3 source-study label(s): (Thakore & Delany, 2025); (Trompet et al, 2024); (Xiong et al, 2025) | Phenotypes: Proliferation (1); Hypertrophy (2); Ossification (3); Growth-plate phenotype (1) Direction: Altered / unclear (5) | In vivo (5); Functional assay (1) | Pathway-causal testing remains limited. (4); Node-level rescue experiments remain limited. (1) | Local signalling nodes converge on ossification, hypertrophy, proliferation; evidence is strongest in vivo but pathway-causal and node-level rescue tests are still limited. |
| Other local signalling readouts | Trpv4; ALP; TRPV4; circRNAs | 3 source-study label(s): (Nevarez et al, 2026); (Liu et al, 2023); (Wu et al, 2022) | Phenotypes: Proliferation (1); Ossification (2); Growth-plate phenotype (3) Direction: Decreased (2); Altered / unclear (3) | Human validation (3); In vivo (3); Association (1) | Pathway-causal testing remains limited. (5) | Local signalling nodes converge on growth-plate phenotype, ossification, proliferation; evidence is strongest in vivo but pathway-causal and node-level rescue tests are still limited. |
| Unspecified local signalling linked to hypertrophy–ossification | Not clearly stated / phenotype-level | 5 source-study label(s): (Zieba et al, 2023); (Costantini et al, 2023); (Xiong et al, 2025); (Zhou et al, 2024); (Trompet et al, 2024) | Phenotypes: Hypertrophy (5); Ossification (5) Direction: Decreased (2); Increased (1); Altered / unclear (2) | Human validation (1); In vivo (4); Association (1) | Pathway-causal testing remains limited. (5) | Local signalling nodes converge on hypertrophy, ossification; evidence is strongest in vivo but pathway-causal and node-level rescue tests are still limited. |
| Unspecified local signalling linked to proliferation | Not clearly stated / phenotype-level | 6 source-study label(s): (Liu et al, 2023); (Zieba et al, 2023); (Baronas et al, 2023); (Zhang et al, 2023); (Saltarelli et al, 2022); +1 more | Phenotypes: Proliferation (5); Hypertrophy (1) Direction: Decreased (1); Increased (1); Impaired (1); Altered / unclear (2) | Human validation (1); In vivo (5) | Pathway-causal testing remains limited. (5) | Local signalling nodes converge on proliferation, hypertrophy; evidence is strongest in vivo but pathway-causal and node-level rescue tests are still limited. |
| Wnt7b signalling | Wnt7b | 1 source-study label(s): (Trompet et al, 2024) | Phenotypes: Hypertrophy (4); Ossification (4) Direction: Decreased (3); Increased (1) | In vivo (1); Functional assay (1); Association (2) | Pathway-causal testing remains limited. (3); Node-level rescue experiments remain limited. (1) | Local signalling nodes converge on hypertrophy, ossification; evidence is strongest in vivo but pathway-causal and node-level rescue tests are still limited. |
| Unspecified local signalling / phenotype-level signal | Not clearly stated / phenotype-level | 2 source-study label(s): (Thakore & Delany, 2025); (Costantini et al, 2023) | Phenotypes: Hypertrophy (1); ECM maturation (1) Direction: Altered / unclear (2) | In vivo (2) | Pathway-causal testing remains limited. (2) | Local signalling nodes converge on hypertrophy, ecm maturation; evidence is strongest in vivo but pathway-causal and node-level rescue tests are still limited. |

**Notes:** This is a synthesis table, not a row-for-row reproduction. Related entries were grouped when they shared route category, molecular axis/readout family, and downstream growth-plate phenotype. Evidence support counts are non-exclusive because a single entry can include more than one support type. **Abbreviations:** ECM, extracellular matrix; GH–IGF, growth hormone–insulin-like growth factor; miR, microRNA; RUNX2, runt-related transcription factor 2; SOX9, SRY-box transcription factor 9; OCN, osteocalcin; OPN, osteopontin; ALP, alkaline phosphatase.

**References**

Ağırdil, Y. (2020) The growth plate: a physiologic overview. *EFORT Open Reviews*, 5(8), 498-507.

Bao, P. L., Jiang, L., Liu, G. L., Li, Y. P., Chen, X. & Wang, M. (2025) Two cases of autosomal dominant familial short stature associated with COL11A2 gene variant and the therapeutic response to recombinant human growth hormone. *Translational Pediatrics*, 14, 618-627.

Baron, J., Sävendahl, L., De Luca, F., Dauber, A., Phillip, M. & Wit, J. M. (2015) Short and tall stature: a new paradigm emerges. *Nature Reviews Endocrinology*, 11(12), 735-746.

Baronas, J. M., Bartell, E., Eliasen, A., Doench, J. G., Yengo, L., Vedantam, S., Marouli, E., Kronenberg, H., Hirschhorn, J. & Renthal, N. E. (2023) Genome-wide CRISPR screening of chondrocyte maturation newly implicates genes in skeletal growth and height-associated GWAS loci. *Cell Genomics*, 3.

Bjarnason, R., Andersson, B., Kim, H., Olsson, B., Swolin-Eide, D. & Wickelgren, R. (2004) Cartilage oligomeric matrix protein increases in serum after the start of growth hormone treatment in prepubertal children. *Journal of Clinical Endocrinology and Metabolism*, 89(10), 5156-5160.

Chen, H., Cui, Y., Li, J., Duan, M., Pi, C., Zhou, X. & Xie, J. (2025) Fibroblast growth factor 19 disrupts cartilage development via the FGFR4/β-catenin axis. *International Journal of Biological Sciences*, 21, 4428-4449.

Chen, Y., Yin, M. Y., Lu, Y., Dong, Z., Lu, W., Lin, L. & Xiao, Y. (2024) Short stature with brachydactyly caused by a novel mutation in the IHH gene and response to 4-year growth hormone therapy: A case report. *Translational Pediatrics*, 13, 856-863.

Cho, J. H., Jung, H. W. & Shim, K. S. (2024) Growth plate closure and therapeutic interventions. *Clinical and Experimental Pediatrics*, 67(11), 553-559.

Chu, N. T. L., Dregval, O., Zaman, F., Li, L., Tian, X., Liu, X. & Chagin, A. S. (2026) A transcriptional atlas of the pubertal human growth plate reveals two populations of stem cells and direct effect of growth hormone. *Science Translational Medicine*.

Costantini, A., Guasto, A. & Cormier-Daire, V. (2023) TGF-β and BMP signaling pathways in skeletal dysplasia with short and tall stature. *Annual Review of Genomics and Human Genetics*, 24, 225-253.

Holling, T., Brylka, L. J., Scholz, T., Bierhals, T., Herget, T., Meinecke, P., Schinke, T., Oheim, R. & Kutsche, K. (2023) TMCO3, a putative K+:proton antiporter at the Golgi apparatus, is important for longitudinal growth in mice and humans. *Journal of Bone and Mineral Research*, 38.

Kang, M. J. (2017) Novel genetic cause of idiopathic short stature. *Annals of Pediatric Endocrinology & Metabolism*, 22(3), 153-157.

Kim, Y. M. (2022) A new era of genetic diagnosis for short stature children: A review. *Precision and Future Medicine*, 6(4), 218-225.

Kim, Y. M., Lim, H. H., Kim, E., Kim, G., Kim, M. & So, H. (2023) Exploring the genetic causes for postnatal growth failure in children born non-small for gestational age. *Journal of Clinical Medicine*, 12(20), 6508.

Kodama, K., Takahashi, H., Oiji, N., Nakano, K., Okamura, T. & Niimi, K. (2020) CANT1 deficiency in a mouse model of Desbuquois dysplasia impairs glycosaminoglycan synthesis and chondrocyte differentiation in growth plate cartilage. *FEBS Open Bio*, 10(6), 1096-1103.

Liu, X., Du, Z., Yi, X., Sheng, T., Yuan, J. & Jia, J. (2021) Circular RNA circANAPC2 mediates the impairment of endochondral ossification by miR-874-3p/SMAD3 signalling pathway in idiopathic short stature. *Journal of Cellular and Molecular Medicine*, 25(7), 3408-3426.

Liu, X., Yan, C., Deng, X. & Jia, J. (2020) Hsa_circularRNA_0079201 suppresses chondrocyte proliferation and endochondral ossification by regulating the microRNA-140-3p/SMAD2 signaling pathway in idiopathic short stature. *International Journal of Molecular Medicine*, 46, 1993-2006.

Liu, X., Yan, C., Deng, X. & Jia, J. (2026) Corrigendum: Hsa_circularRNA_0079201 suppresses chondrocyte proliferation and endochondral ossification by regulating the microRNA-140-3p/SMAD2 signaling pathway in idiopathic short stature. *International Journal of Molecular Medicine*, 57.

Liu, X., Yuan, J., Wu, Z., Zhang, J., Shen, Y. & Jia, J. (2023) Plasma exosome miRNA-26b-3p derived from idiopathic short stature impairs longitudinal bone growth via the AKAP2/ERK1/2 axis. *Journal of Nanobiotechnology*, 21.

Mongiat, M., Taylor, K., Otto, J., Aho, S., Uitto, J., Whitelock, J. M. & Iozzo, R. V. (2000) The protein core of the proteoglycan perlecan binds specifically to fibroblast growth factor-7. *J Biol Chem*, 275(10), 7095-7100.

Nevarez, L., Ismaili, T., Zieba, J. T., Martin, J. H., Wachtell, D., Diaz, D., Ramirez, J. A., Aceves, V., Ito, J., Gray, R. S., Goldstein, D. B., Sahdeo, S., Krakow, D. & Cohn, D. H. (2026) Small molecule inhibition rescues the skeletal dysplasia phenotype of Trpv4 mutant mice. *JCI Insight*, 11.

Ono, K., Karolak, M. R., Ndong, J. D., Wang, W., Yang, X. & Elefteriou, F. (2013) The ras-GTPase activity of neurofibromin restrains ERK-dependent FGFR signaling during endochondral bone formation. *Human Molecular Genetics*, 22(15), 3048-3062.

Pan, Y., Zhang, F., Zhong, J., Gong, Q., Geng, N., Kuang, B., Lan, Q., Yi, M., Zeng, Q., Chen, C., Fan, M., Zhao, C., Zeng, D., Du, Y., Nie, M., Wang, Z. & Guo, F. (2026) CircTspan3 promotes cartilage development through ANNEXIN A2-mediated ferroptosis and apoptosis inhibition and exosome-mediated paracrine signaling. *Advanced Science*, 13.

Paparella, R., Bei, A., Bernabei, I., Tarani, F., Niceta, M. & Pucarelli, I. (2025) Idiopathic short stature in the genomic era: Integrating auxology, endocrinology, and emerging genetic insights. *Children*, 12(7), 855.

Rashid, H., Smith, C. M., Convers, V., Clark, K. & Javed, A. (2024) Runx2 deletion in hypertrophic chondrocytes impairs osteoclast mediated bone resorption. *Bone*.

Saltarelli, M. A., Quarta, A. & Chiarelli, F. (2022) Growth plate extracellular matrix defects and short stature in children. *Annals of Pediatric Endocrinology & Metabolism*, 27, 247-255.

Shin, M., Kim, H., Ryu, S. & Kim, S. H. (2026) Circulating exosomal microRNA profiles associated with heavy metal exposure and short stature in children. *International Journal of Molecular Sciences*, 27.

Starrett, J. H., Lemoine, C., Guillo, M., Fayad, C., Kaci, N., Neal, M., Pettitt, E., Pache, M., Ye, Q., Chouinard, M., Allen, E. L., Baujat, G., Hudkins, R. L., Bober, M. B., Harris, T., Swanson, R. V. & Legeai-Mallet, L. (2025) TYRA-300, an FGFR3-selective inhibitor, promotes bone growth in two FGFR3-driven models of chondrodysplasia. *JCI Insight*, 10.

Taieb, M., Ghannoum, D., Barré, L. & Ouzzine, M. (2023) Xylosyltransferase I mediates the synthesis of proteoglycans with long glycosaminoglycan chains and controls chondrocyte hypertrophy and collagen fibers organization in the growth plate. *Cell Death & Disease*, 14.

Taye, N., Karoulias, S. Z., Balic, Z., Wang, L. W., Willard, B. B. & Martin, D. (2025) Combined ADAMTS10 and ADAMTS17 inactivation exacerbates bone shortening and skin phenotypes. *Life Science Alliance*, 8(12), e202503232.

Thakore, P. & Delany, A. M. (2025) miRNA-based regulation in growth plate cartilage: mechanisms, targets, and therapeutic potential. *Frontiers in Endocrinology*, 16, 1530374.

Trompet, D., Kurenkova, A. D., Zhou, B., Li, L., Dregval, O., Usanova, A. P., Chu, T. L., Are, A., Nedorubov, A. A., Kasper, M. & Chagin, A. (2024) Stimulation of skeletal stem cells in the growth plate promotes linear bone growth. *JCI Insight*, 9.

Tsukamoto, S., Kuratani, M., Tanaka, S., Jimi, E., Oda, H. & Katagiri, T. (2023) Wnt7b expressed by hypertrophic chondrocytes is a stimulatory factor for endochondral ossification that is regulated by Smad4 activity. *Development*, 150(15).

Wang, Y., Wu, Z., Yuan, J., Zhu, J., Ventin, M. & Arya, S. (2025) Capsaicin diet drives gut inflammation and exosomal miR-17-3p elevation in idiopathic short stature. *Nature Communications*, 17(1), 1123.

Weaver, S. R., Peralta-Herrera, E., Torres, H. M., Jessen, E., Bradley, E. W. & Westendorf, J. J. (2024) Phlpp1 alters the murine chondrocyte phospho-proteome during endochondral bone formation. *Bone*.

Whitaker, A. T., Berthet, E., Cantú, A. V., Laird, D. J. & Alliston, T. (2017) Smad4 regulates growth plate matrix production and chondrocyte polarity. *Biology Open*, 6(3), 358-364.

Wu, Z., Yuan, J., Li, J., Du, Z., Yin, M., Cheng, X., Liu, X. & Jia, J. (2022) Hsa_circ_0008870 suppresses bone formation of growth plate through inhibition of miR-185-3p/MAPK1 axis in idiopathic short stature. *Frontiers in Bioengineering and Biotechnology*, 10.

Xiong, J., Ma, R., Xie, K., Shan, C., Chen, H., Wang, Y., Liao, Y., Deng, Y., Ye, G., Wang, Y., Zhu, Q., Zhang, Y., Cai, H., Guo, W., Yin, Y. & Li, Z. (2025) Recapitulation of endochondral ossification by hPSC-derived SOX9+ sclerotomal progenitors. *Nature Communications*, 16.

Yokoyama, Y., Kameo, Y., Sunaga, J., Maki, K. & Adachi, T. (2024) Chondrocyte hypertrophy in the growth plate promotes stress anisotropy affecting long bone development through chondrocyte column formation. *Bone*.

Yu, X. F., Teng, B., Li, J., Zhang, J. V., Su, Z. & Ren, P. G. (2024) Novel function of osteocalcin in chondrocyte differentiation and endochondral ossification revealed on a CRISPR/Cas9 bglap–bglap2 deficiency mouse model. *International Journal of Molecular Sciences*, 25.

Yuan, J., Du, Z., Wu, Z., Yang, Y., Cheng, X. & Liu, X. (2021) A novel diagnostic predictive model for idiopathic short stature in children. *Frontiers in Endocrinology*, 12, 721812.

Yuan, J., Guo, L., Wang, J., Zhou, Z. & Wu, C. (2023) α-Parvin controls chondrocyte column formation and regulates long bone development. *Bone Research*, 11.

Yuan, J., Wang, Y., Huang, Y., Li, S., Zhang, X., Wu, Z. & Jia, J. (2024) Investigating novel therapeutic approaches for idiopathic short stature: Targeting siRNA and growth hormone delivery to the growth plate using exosome nanoparticles. *Advanced Science*, 11.

Zhang, J., Du, L., Davis, B., Gu, Z., Lyu, J., Zhao, Z., Xu, J. & Morrison, S. J. (2023) Osteolectin increases bone elongation and body length by promoting growth plate chondrocyte proliferation. *Proceedings of the National Academy of Sciences of the United States of America*, 120.

Zhang, Y., Wen, J., Lai, R., Zhang, J., Li, K., Zhang, Y., Liu, A. & Bai, X. (2024) Rheb1 is required for limb growth through regulating chondrogenesis in growth plate. *Cell and Tissue Research*, 395, 261-269.

Zhou, Z., Mao, X., Jiang, C., Li, W., Zhou, T., Liu, M., Sun, S., Wang, M., Dong, N., Wu, Q. & Zhou, H. (2024) Deficiencies in corin and atrial natriuretic peptide-mediated signaling impair endochondral ossification in bone development. *Communications Biology*, 7.

Zieba, J. T., Nevarez, L., Wachtell, D., Martin, J. H., Kot, A., Wong, S., Cohn, D. H. & Krakow, D. (2023) Altered Sox9 and FGF signaling gene expression in Aga2 OI mice negatively affects linear growth. *JCI Insight*, 8.
